# StableTi_3_C_2_T*_x_* MXene Ink Formulation and High‐Resolution Aerosol Jet Printing for High‐Performance MXene Supercapacitors

**DOI:** 10.1002/smtd.202500499

**Published:** 2025-05-28

**Authors:** Fereshteh Rajabi Kouchi, Tony Valayil Varghese, Hailey Burgoyne, Naqsh E Mansoor, Myeong‐Lok Seol, Nicholas McKibben, Shruti Nirantar, Karthik Chinnathambi, Josh Eixenberger, Olivia Maryon, Christopher E Shuck, Yury Gogotsi, Jessica E. Koehne, David Estrada

**Affiliations:** ^1^ Micron School of Materials Science and Engineering Boise State University Boise ID 83725 USA; ^2^ NASA Ames Research Center Universities Space Research Association Moffett Field CA 94035 USA; ^3^ School of Engineering RMIT University Melbourne VIC 3001 Australia; ^4^ Departmentof Physics Boise State University Boise ID 83725 USA; ^5^ Center for Advanced Energy Studies Boise State University Boise ID 83725 USA; ^6^ A.J. Drexel Nanomaterials Institute and Department of Materials Science and Engineering Drexel University Philadelphia PA 19104 USA; ^7^ Department of Chemistry and Chemical Biology Rutgers University Piscataway NJ 08854 USA; ^8^ Idaho National Laboratory Idaho Falls ID 83415 USA

**Keywords:** additive manufacturing, aerosol jet printing, high‐resolution printing, ink formulation, MXene supercapacitor

## Abstract

Lightweight energy storage devices are essential for developing compact wearable and distributed electronics, and additive manufacturing offers a scalable, low‐cost approach to fabricating such devices with complex geometries. However, additive manufacturing of high‐performance, on‐demand energy storage devices remains challenging due to the need for stable, multifunctional nanomaterial inks. Herein, the development of 2‐dimensional (2D) titanium carbide (Ti_3_C_2_T*_x_* MXene) ink that is compatible with aerosol jet printing for energy storage applications is demonstrated. The developed MXene ink demonstrates long‐term chemical and physical stability, ensuring consistent printability and achieving high‐resolution prints (≈45 µm width lines) with minimal overspray. The high‐resolution aerosol‐jet printed MXene supercapacitor achieves an areal capacitance of 122 mF cm^−2^ and a volumetric capacitance of 611 F cm^−3^, placing them among the highest‐performing printed supercapacitors reported to date. These findings highlight the potential of aerosol jet printing with MXene inks for on‐demand, scalable, and cost‐effective fabrication of printed electronic and electrochemical devices.

## Graphical abstract

A novel additive free Ti_3_C_2_T*_x_* MXene ink is formulated for aerosol jet printing, enabling stable, high‐resolution patterning. The printed micro‐supercapacitors deliver strong electrochemical performance, illustrating the use of additive manufacturing for compact energy storage in wearable and distributed electronics.

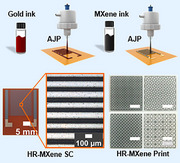

## 1 Introduction

Energy storage devices, such as rechargeable batteries and supercapacitors, play a crucial role in the development of portable and wearable electronics, hybrid electric vehicles, and smart grids.^[^ ^1^, ^2^, ^3^, ^4^, ^5^ ^]^ While batteries are well‐known for their high energy density, their charge/discharge rates and cycle life are often limited, restricting their use in applications requiring high operational stability or rapid charge/discharge rates. In contrast, supercapacitors offer high power density, long operational lifetime, and a rapid charge‐discharge rate, making them a promising candidate for the electrification of energy infrastructure, portable electronics, and electric vehicles.^[^ ^6^, ^7^, ^8^ ^]^ Based on the charge storage mechanisms, supercapacitors are characterized into electrical double‐layer capacitors (EDLC) and pseudocapacitors. As indicated by the name, EDLCs store charge due to the formation of an electrical double layer induced by electrostatic forces that attract electrolyte ions to the surface of the electrode, whereas, pseudocapacitors store charge by Faradic charge transfer between the electrode and the electrolyte.^[^ ^9^, ^10^, ^11^ ^]^ The choice of the electrode material is critical for optimizing supercapacitor performance, influencing parameters such as capacitance, power density, and cycling stability.

Recently research has focused on identifying novel 2D nanomaterials that can offer various charge storage mechanisms. Among those, 2D transition metal carbides, nitrides, and carbonitrides, known as MXenes, have demonstrated superior performance.^[^ ^12^ ^]^ The general formula of MXenes is M*_n_*_+1_X*_n_* T*_x_* (*n* = 1–4), where M is an early transition metal (Ti, Nb, V, etc.), X represents carbon and/or nitrogen, and T*_x_* refers to the surface functional groups (primarily ─O, ─F, or ─OH) terminations.^[^ ^13^, ^14^ ^]^ MXenes have emerged as promising electrode materials for electrochemical energy storage applications due to their unique structure, with an inner conductive transition metal carbide layer, efficient electron transportation, variable hydrophilic functional groups that serve as active sites for rapid redox reactions, and lamellar structure that provides easy ion intercalation between 2D layers.^[^ ^15^, ^16^ ^]^ The hydrophilic surface functional groups and negative surface charge of the MXene flakes allow MXenes to form stable colloidal dispersion in various aqueous and organic solvents, making MXenes excellent materials for solution processing and additive manufacturing.^[^ ^14^ ^]^

Compared to conventional microfabrication processes, novel printing technologies offer rapid, high‐volume device fabrication on a wide variety of substrates with minimal material wastage.^[^ ^17^, ^18^, ^19^, ^20^, ^21^, ^22^, ^23^ ^]^ Manufacturing supercapacitors using additive manufacturing combines their remarkable stability and durability with the aforementioned benefits of printing.^[^ ^24^ ^]^ However, significant enhancement in device performance can be achieved through careful patterning and controlled porosity, which can further expand printed supercapacitor applications toward in‐space manufacturing missions, forward‐deployed defense missions, and roll‐to‐roll manufacturing for commercial systems.^[^ ^25^ ^]^

While many advances have been made in the solution processing of 2D materials, developing suitable and printable functional inks remains challenging and requires careful consideration before use in electronic device fabrication.^[^ ^26^, ^27^, ^28^ ^]^ MXenes can be readily dispersed in water, such dispersions are highly susceptible to oxidation and are typically degrade within a few days at room temperature (≈30 days).^[^ ^29^ ^]^ Studies have demonstrated that this degradation can be mitigated by altering the dispersion medium,^[^ ^30^, ^31^ ^]^ incorporating antioxidants or polymers,^[^ ^30^, ^31^, ^32^ ^]^ utilizing organic solvents,^[^ ^33^ ^]^ or employing water purged with argon to reduce oxygen exposure.^[^ ^34^ ^]^ Each printing technique demands specific fluidic and rheological properties.^[^ ^27^ ^]^ Achieving the required viscosity, surface tension, and drying behavior without sacrificing MXene stability or adding performance‐limiting surfactants is a significant challenge. While additives and surfactants can improve ink rheology, they often require post‐treatment for removal—typically involving high temperatures that may damage MXene flakes and impair device performance. Similarly, organic solvents like N‐methyl‐2‐pyrrolidone (NMP) and dimethyl sulfoxide (DMSO),^[^ ^28^, ^35^ ^]^ which have high boiling points and toxicity, making them less suitable for large‐scale additive manufacturing. Therefore, there is lack of stable, additive‐free MXene inks that offer both long shelf‐life and necessary rheological and drying characteristics for high‐resolution and high‐performance device fabrication.

Inkjet printing (IJP) has been the primary candidate for printing nanomaterial inks, offering digital, maskless patterning, non‐contact deposition, high spatial resolution, and compatibility with roll‐to‐roll manufacturing. However, it poses challenges in developing printable inks with appropriate rheological, fluidic, and morphological properties.^[^ ^36^ ^]^ Screen printing is widely recognized for its low‐cost, high‐throughput, and scalable nature, enabling the printing of thick, uniform films with tunable thickness. It is well‐suited for printable formulations with high viscosity, and its non‐Newtonian fluid compatibility supports continuous deposition of functional inks. Similarly, extrusion‐based printing allows for the deposition of highly viscous, shear‐thinning inks, often in the form of pastes or gels, and enables the fabrication of 3D structures or thick, patterned films. This makes it particularly attractive for energy storage applications like supercapacitors. However, both screen and extrusion‐based printing share common limitations, including low spatial resolution, high roughness, and the requirement for high material loading to make a printable paste. In addition, the ink formulation involves the addition of polymeric binders and modifiers due to the requirements of high ink viscosities, necessitating additional post‐printing processes to enhance functionality which can affect the performance of the device.^[^ ^19^, ^37^ ^]^

Recently, aerosol jet printing (AJP) has emerged as a powerful tool in additive electronics manufacturing, offering non‐contact, high‐resolution prints, mask‐less design capabilities, and compatibility with a wide viscosity range (≈1 to 1000 cP) exceeding conventional inkjet printing systems.^[^ ^38^, ^39^, ^40^, ^41^, ^42^ ^]^ These features make AJP a suitable manufacturing technique for developing various nanomaterial‐based electronic devices onto flexible and rigid substrates.^[^ ^43^, ^44^, ^45^ ^]^ Previous studies have reported printing MXene supercapacitors (SCs) using inkjet printing, screen printing, and extrusion‐based 3D printing.^[^ ^25^, ^35^, ^46^ ^]^ However, these methods suffer from low printing resolution and require binders and additives, requiring additional post‐processing to achieve device performance.^[^ ^19^, ^37^ ^]^

**Figure 1:**
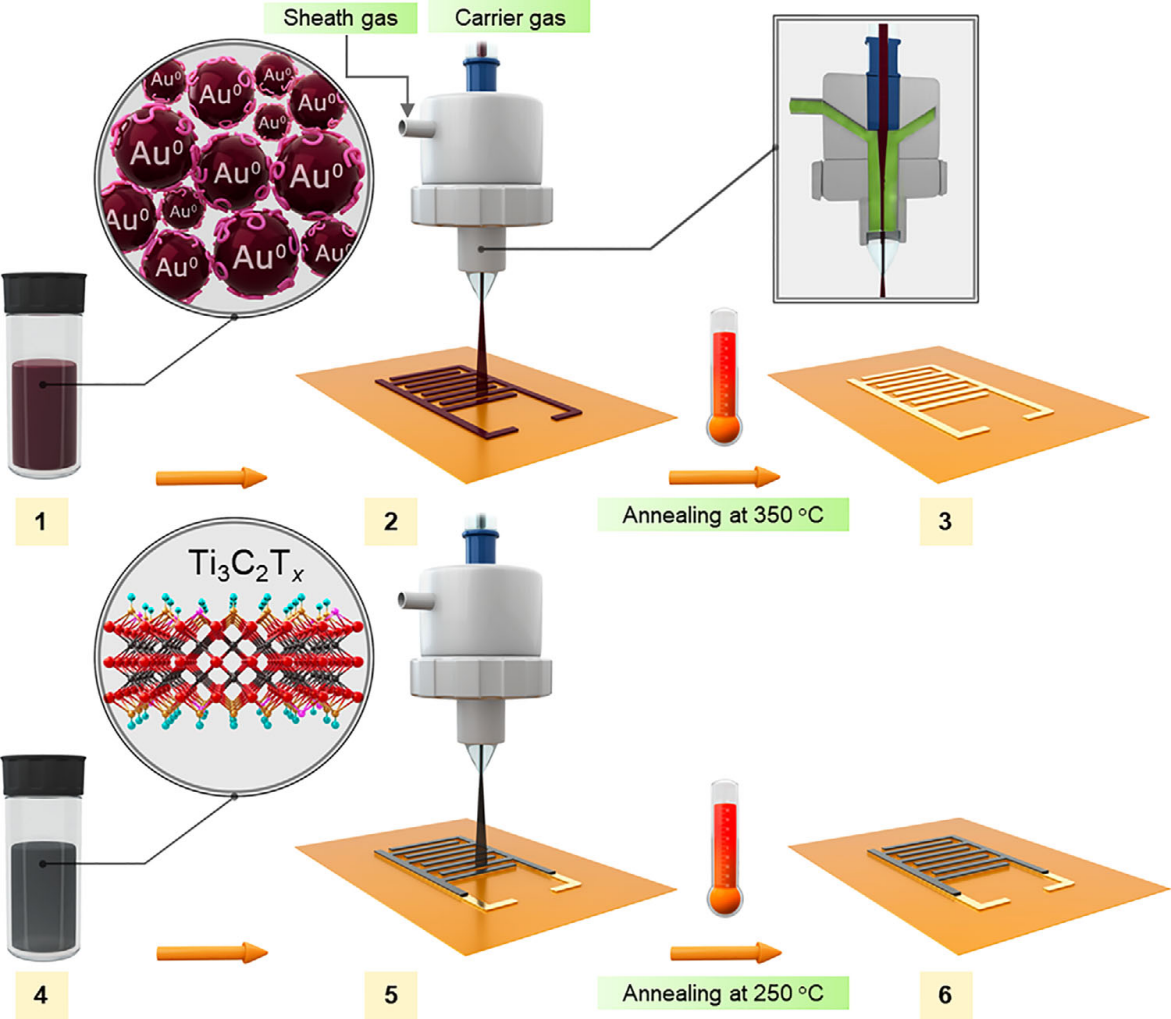
Schematic illustration of aerosol jet printing of MXene supercapacitor on polyimide. 1) Gold nanoparticle ink was prepared based on our previous work^65^. 2) Aerosol jet printing of gold ink on polyimide substrate as a current collector. 3) Annealing printed gold at 350 °C. 4) Formulating Ti_3_C_2_T*_x_* MXene ink. 5) Aerosol jet printing of MXene ink on top of the annealed gold current collector. 6: Annealing printed SCs at 250 °C for 2 h under Ar/N_2_ gas.

Here, we develop a chemically and physically stable, polymer/surfactant‐free Ti_3_C_2_T*_x_* MXene ink compatible with AJP (**Figure** **1**). The formulated ink delivers consistent printing performance, achieving high‐resolution prints with minimal overspray and coffee ring issues. High resolution with a line width of ≈45 µm and complex patterns were successfully printed on sapphire and alumina tubes, demonstrating the planar and conformal printability of AJP. To assess the electrical properties of Ti_3_C_2_T*_x_*, we use the formulated ink for AJP of Ti_3_C_2_T*_x_* lines with gold contact pads in a transmission line measurements (TLM) structure. The microscale porosity, pore size, and pore morphology of printed MXene lines on a silicon substrate were studied using a novel Sauvola method.^[^ ^47^ ^]^ This analysis provides insight into the porosity of printed devices and its influence on overall performance. Aerosol jet printing was employed for depositing gold nanoparticles as the current collectors and Ti_3_C_2_T*_x_* MXene as an active electrode material as shown in (Figure 1). The aerosol jet printed MXene SCs exhibit high areal capacitance and volumetric capacitance, with MXene thickness as small as ≈1.25 µm, enabling the fabrication of high‐resolution printed supercapacitors. This work highlights the potential of employing AJP with stable MXene ink formulations for the scalable manufacturing of micro‐supercapacitors, sensors, and electronic and energy harvester devices.

## 2 Results

### 2.1 Production of Ti_3_C_2_T_x_ MXene Nanoflakes

We synthesize large multilayer (*ml*)‐Ti_3_C_2_T*_x_* MXene flakes with few defects using the minimally intensive layer delamination (MILD) etching method, as detailed in the Experimental Section. This method eliminates the need for sonication, as delamination naturally occurs during the washing step, eliminating the need for additional post‐treatment.^[^ ^48^ ^]^ To formulate Ti_3_C_2_T*_x_* ink with the desired particle size, the synthesized (*ml*)‐Ti_3_C_2_T*_x_* powder underwent sonication to reduce particle size, ensuring compatibility with AJP. Notably, smaller MXene flakes suffer from decreased electrical conductivities due to increased interfacial contact resistance, but at the same time, they provide more accessible ion diffusion paths, enhancing the electrochemical performance of the printed structure.^[^ ^49^ ^]^ This trade‐off between electrical conductivity and ion accessibility is a critical factor in optimizing the performance of printed MXene supercapacitors (SCs). We employed sonication to achieve compatibility with AJP and incorporated a gold current collector to enhance the conductivity. The combination of these approaches resulted in improved electrochemical performance, effectively balancing conductivity and the ion diffusion pathway.

Structural analysis was performed via X‐ray powder diffraction (XRD) for the Ti_3_AlC_2_ MAX phase and the synthesized (*ml*)‐Ti_3_C_2_T*_x_*. Figure S1a (Supporting Information) shows the XRD patterns of Ti_3_AlC_2_ MAX phase and (*ml*)‐Ti_3_C_2_T*_x_*. Three characteristic peaks of the MAX phase are observed at 2θ = 9.45°, 19.03°, and 38.92°, corresponding to the (002), (004), and (104) planes, respectively. The disappearance of the non‐basal peak at 2θ = 38.92° and the increase in the relative intensity of the (002) peak indicate the complete etching of aluminum from the MAX phase. Additionally, the shift of the (002) peak to a lower 2θ, from 9.45° to 7.37°, corresponds to an increase in the *c*‐lattice parameter (*c*‐LP) constant after the removal of the aluminum atomic layer, expanding the *d*‐spacing from 9.36 to 12 Å.^[^ ^50^ ^]^ The SEM images of Ti_3_AlC_2_ MAX phase and (*ml*)‐Ti_3_C_2_T*_x_* MXene (Figure S1b,c, Supporting Information) show morphological changes after etching aluminum from the MAX phase, resulting in an expanded (*ml*)‐Ti_3_C_2_T*_x_* layered structure.

Figure S1d (Supporting Information) shows the X‐ray photoelectron spectroscopy (XPS) survey spectrum of (*ml*)‐Ti_3_C_2_T*_x_* MXene, which gives signals mainly for C 1s, Ti 2p, O 1s, F 1s, and Cl 2p with the atomic percentage of 36.3%, 26.6%, 18.9%, 13.6%, and 4.6% respectively and the absence of Al peak confirms the complete removal of the Al layers during the synthesis process. To further investigate the chemical structure modifications of the (*ml*)‐MXene, core level XPS spectra of Ti 2p and C 1s were analyzed (Figure S1e,f, Supporting Information). The core level Ti 2p spectrum was fitted with four doublets. The Ti 2p_3/2_ peaks centered at ≈455.1, 456.04, and 457.0 eV, corresponding to C─Ti─(O/O/O), C─Ti─(O/O/F), and C─Ti─(O/F/F), respectively as in previous reports.^[^ ^51^ ^]^ In addition, the peak located at ≈459.6 eV represents TiO_2‐*x*_F_2x_. The high‐resolution C 1s spectrum was fitted with three components at 282.0, 284.4, and 286.5 eV attributed to Ti─C─Ti, C─C, and C─O, indicating a negligible amounthe t of TiO_2_ in the synthesized (*ml*)‐MXene using the MILD method. In addition, the (*ml*)‐MXene flakes are terminated with F, Cl, and OH functional groups.

The particle size of the obtained (*ml*)‐Ti_3_C_2_T*_x_* powder after the acid etching was larger than 4 µm (Figure S1c, Supporting Information). Large particle sizes can lead to nozzle clogging during the AJP process. In order to avoid nozzle clogging, the particle size is generally kept smaller than 1/50 of the nozzle diameter.^[^ ^52^ ^]^ Probe sonication was employed to reduce the flake size of (*ml*)‐Ti_3_C_2_T*_x_*, ensuring compatibility with the AJP process.

The solvent choice plays a critical role in probe sonication for size reduction and exfoliation of 2D materials.^[^ ^53^, ^54^ ^]^ Polar solvents such as water, DMSO, dimethylformamide (DMF), and NMP are suitable solvents for O─, OH─ and F─terminated MXenes, since their polarity and surface tension of the solvent matches the polarity and surface energy of the MXene.^[^ ^53^ ^]^ In addition, using organic‐based solvents reduces the probability of oxidization and maintains the electronic properties of the MXene. Therefore, the (*ml*)‐Ti_3_C_2_T*_x_* powder was initially dispersed into NMP solvent at a concentration of 15 mg mL^−1^, followed by probe sonication for 9 h at a power of 420 W (60% amplitude).

**Figure 2:**
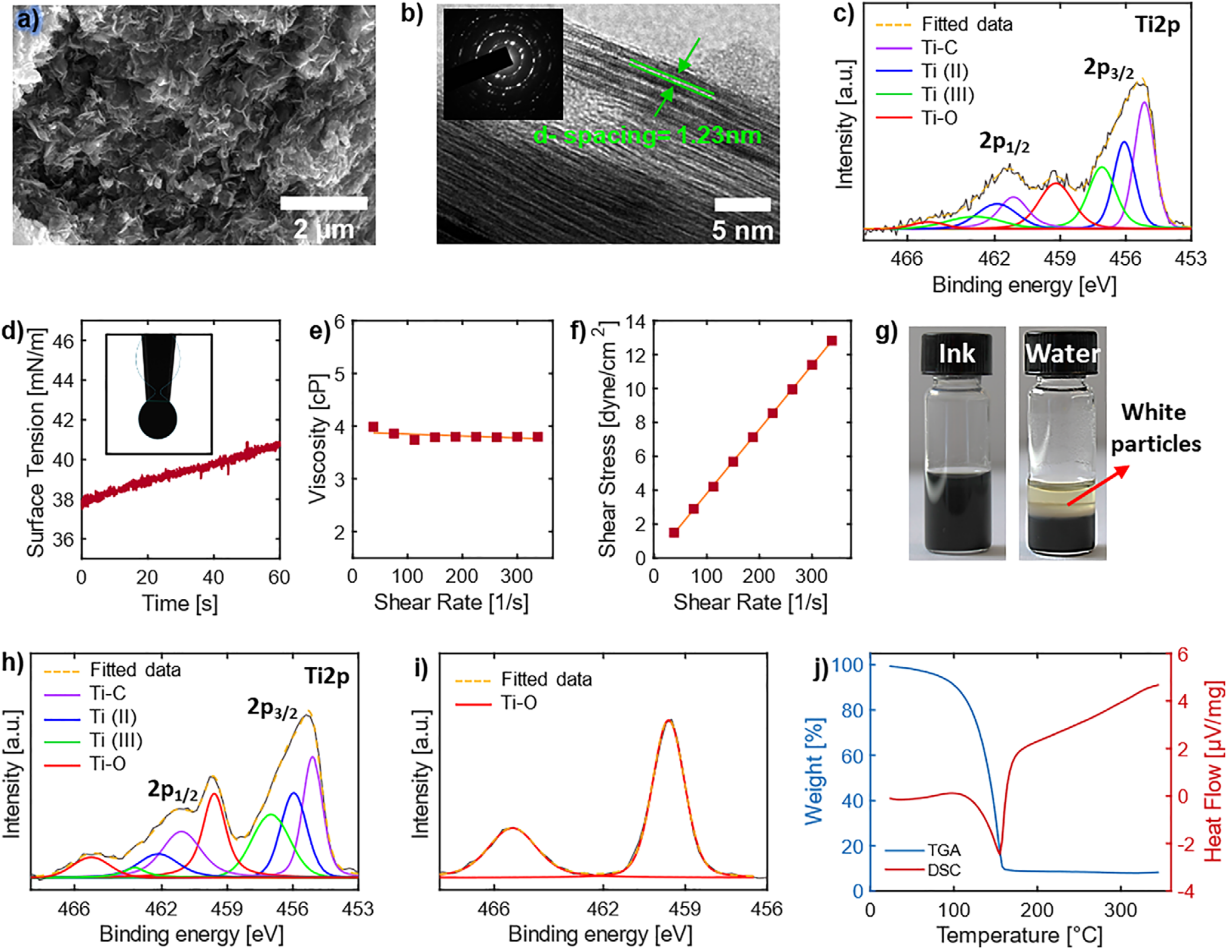
Material and ink characterization. a) Scanning electron microscopy (SEM) image of exfoliated Ti_3_C_2_T*_x_* MXene. b) Transmission electron microscopy (TEM) of exfoliated Ti_3_C_2_T*_x_* MXene with the selected area electron diffraction (SAED) in the inset, showing the layered structure of MXene with *d*‐spacing of 1.23 nm. c) High‐resolution XPS spectra of Ti 2p for exfoliated Ti_3_C_2_T_x_ MXene. d) Surface tension study of formulated Ti_3_C_2_T*_x_* ink over 60 s. e) Viscosity versus shear rate plot showing a linear relation, confirming the Newtonian behavior of the ink. f) Linear relation between shear stress and shear rate. g) Optical images comparing the stability of the MXene ink (left) and MXene nanosheets dispersed in water (right), both at a concentration of 25 mg mL^−1^, after six months. The presence of a white layer on top of the water dispersion indicates the oxidization of MXene nanosheets. h) High‐resolution XPS spectra of Ti 2p for Ti_3_C_2_T*_x_* MXene ink after six months (concentration of 25 mg mL^−1^), showing chemical stability. i) High‐resolution XPS spectra of Ti 2p for exfoliated Ti_3_C_2_T*_x_* MXene dispersed in water after six months (concentration of 25 mg mL^−1^), highlighting changes due to oxidation. j) Thermal analysis (TGA‐DSC) of MXene ink, used to determine the optimal annealing temperature for the post‐printing process.

While Ti_3_C_2_T*_x_* can be dispersed in various polar organic solvents, including NMP, its high boiling point, and toxicity make it unsuitable for additive manufacturing applications. Although previous studies have reported the printing of MXene inks using NMP and DMSO as the solvent, they have not sufficiently addressed the toxicity concerns associated with operator exposure to the solvent system during the fabrication process.^[^ ^28^, ^35^ ^]^ In addition, a comprehensive study on the formulation of MXene ink specifically for aerosol jet printing of high‐resolution devices is currently lacking. To address these limitations, a solvent exchange process was implemented to re‐formulate MXene inks into a co‐solvent system that is less toxic and ensures consistent printability. Following exfoliation and centrifugation, the supernatant was collected and introduced into an aqueous NaCl solution to facilitate the flocculation of Ti_3_C_2_T*_x_* flakes from NMP, which was used only for the exfoliation process. **Figure** **2**a presents an SEM image of exfoliated MXene sheets, revealing a transparent flake‐like structure of Ti_3_C_2_T*_x_* nanoflakes with a size reduction to less than 1 µm, indicating effective exfoliation. Figure 2b shows a TEM image of exfoliated few‐layer Ti_3_C_2_T*_x_*, with the selected area electron diffraction (SAED) shown in the inset. It features a typical 2D multi‐layered structure, consisting of three layers of titanium atoms with two layers of carbon atoms, and an interlayer spacing of 1.23 nm along the *c*‐direction, validating the previously discussed *d*‐spacing increase. Bright dots in the SAED pattern exhibit a hexagonal symmetry, as demonstrated in the inset. Statistical analysis of atomic force microscopy (AFM) imaging (Figure S2a–e, Supporting Information) further confirmed the lateral size and thickness distribution of nanoflakes, showing lateral dimensions less than 400 nm and thickness less than 100 nm, confirming the size reduction after probe sonication.

Figure S2f (Supporting Information) shows XPS spectra of exfoliated Ti_3_C_2_T*_x_* MXene, which mainly gives signals C 1s, Ti 2p, O 1s, F 1s, Cl 2p, and N 1s with the atomic percentage of 34.4, 25.35, 22.0, 14, 6.6, and 1.8% respectively. The small nitrogen peak at ≈398–401 eV for exfoliated MXene indicates the presence of trapped and adsorbed NMP molecules resulting from the size reduction step. The high‐resolution Ti 2p peaks indicate that there is a negligible increase in the intensity of the TiO_2_ peak (Figure 2c). In addition, the deconvoluted C 1s cove‐level spectra (Figure S2g, Supporting Information) for exfoliated MXene show that a negligible change in C─O contribution compared to the (*ml*)‐MXene. Therefore, both Ti 2p and C 1s cove‐level spectra confirm the advantage of utilizing an organic solvent in solution processing step to avoid oxidation of MXene nanoflakes.

### 2.2 Formulation of Ti_3_C_2_T_x_ MXene Ink

Developing a printable ink suitable for AJP requires careful consideration of various parameters, including solvent/co‐solvent vapor pressure, ink rheological properties, and stability of colloidal solutions.^[^ ^55^, ^56^ ^]^ Understanding the rheological properties of the inksuch as viscosity, surface tension, density, and particle size—is crucial for determining printability, atomization yield, droplet size distribution in the aerosolized ink, and ultimately printing resolution.^[^ ^57^ ^]^ A primary solvent with moderate volatile and low viscosity (less than 10 cP) enhances atomization yield and improves printing resolution.^[^ ^58^, ^59^ ^]^ The negative electrostatic charge on hydrophilic Ti_3_C_2_T*_x_* MXene flakes (with a Zeta potential of −30 to 80 mV)^[^ ^14^ ^]^ eliminates the need for further treatment or additives to achieve a stable dispersion in aqueous or hydrophilic solvents. Dispersed Ti_3_C_2_T*_x_* MXene in water is both cost‐effective and environmentally friendly, and water exhibits suitable properties, including a moderate evaporation rate that simplifies handling and device fabrication steps. However, it is worth noting that the surface tension of water is 72 mN m^−1^, exceeding the recommended value by AJP manufacturers,^[^ ^60^ ^]^ which can lead to decreased atomization yield and monodispersibility of droplet size, potentially causing overspray, inconsistent printing, and reduced printing resolution.^[^ ^60^ ^]^ Maleski et al. reported that achieving a stable colloidal solution with Ti_3_C_2_T*_x_* may require solvents with surface tension higher than 40 mN m^−1^. Moreover, despite its notably lower surface tension, ethanol has been identified as a suitable solvent for MXene dispersion due to its strong hydrogen bonding with MXene surface functional groups, making it a suitable solvent for MXenes dispersions.^[^ ^53^ ^]^ Ti_3_C_2_T*_x_* MXene flakes maintain a stable dispersion in both high‐ and low‐viscosity solvents, highlighting the importance of selecting an appropriate solvent viscosity based on the specific application. For AJP utilizing an ultrasonic atomizer, an ink with a viscosity of less than 10 cP is ideal to ensure optimal performance.

To formulate an MXene ink, exfoliated Ti_3_C_2_T*_x_* powder was dispersed in a co‐solvent system consisting of water, ethanol, and ethylene glycol (5:4:1 ratio) at a concentration of 25 mg mL^−1^. Water and ethanol were incorporated to adjust the surface tension of the ink and improve the drying behavior of the ink by creating a surface tension gradient during the drying process. The hydrogen interaction between water and ethanol with hydrophilic functional groups on MXene contributes to enhanced ink stability and dispersibility. Ethylene glycol was used to fine‐tune the viscosity of the ink to ensure optimal printability. The rheological properties of Ti_3_C_2_T*_x_* dispersion in the co‐solvent ink were extensively studied (Figure 2d–f). The surface tension‐time plot (Figure 2d) indicates that the ink has a surface tension of 38–40 mN m^−1^ over 60 s. The slight increase in surface tension over time corresponds to the evaporation of ethanol with lower surface tension (≈23 mN m^−1^). Figure 2e,f show the viscosity‐shear rate and the shear stress‐shear rate plots respectively, confirming the Newtonian characteristics of the formulated ink, with a viscosity (η) of 3.8 cP, suitable for ultrasonic atomization. Additionally, the Ti_3_C_2_T*_x_* MXene flakes present a Zeta potential of 28–38 mV in the co‐solvent system, indicating good colloidal stability.

Figure S3a,b (Supporting Information) show photographs of the Ti_3_C_2_T*_x_* dispersions in two media: a co‐solvent system (water, ethanol, and ethylene glycol) and water, respectively. These images were taken every month using a digital camera to monitor the dispersion stability of the developed ink and MXene suspension in water. Both solutions had a concentration of 5 mg mL^−1^, a sufficiently low concentration to facilitate optical monitoring of colloidal stability. The MXene dispersion in water began to precipitate after one month, whereas the co‐solvent system maintained colloidal stability for up to three months. This high physical stability of the ink correlates to the proper surface tension, viscosity, and hydrogen bonding interactions, which effectively interact with functional groups on MXene flakes.^[^ ^53^ ^]^

In addition, a higher concentration (25 mg mL^−1^) of the MXene ink was prepared and compared to MXene dispersed in water at the same concentration (Figure 2g). After six months, a white oxidized layer formed on top of the dispersed MXene in water, indicating significant MXene degradation. In contrast, the ink did not show this oxidized layer which was further validated with XPS, confirming enhanced chemical stability in the co‐solvent system. Therefore, the formulated ink is stable not only in terms of dispersibility but also in terms of reactivity compared to dispersed MXene nanosheets in water. Introducing other solvents can reduce the influence of water to MXene and hinder oxidation. We hypothesize that since the water content in the ink is reduced to half compared to that in pure aqueous dispersions, and the presence of polar ethanol and ethylene glycol limits the access of water to the surface and edges of MXene flakes by the formation of hydrogen bonds with surface terminal groups of MXenes flakes, therefore increasing the ink chemical stability of the formulated ink. Moreover, ethylene glycol, which exhibits lower oxygen solubility compared to water and ethanol, has been reported to play a significant role in reducing the oxidation of MXenes in solution, further enhancing the long‐term chemical stability of the ink.^[^ ^61^, ^62^ ^]^

While the high concentration of the ink made it difficult to visually observe particle settling of MXene nanosheets, minor sedimentation was observed at the bottom of the container after six months. XPS analysis was conducted to further study the chemical composition of both solutions after six months. Figure S3c,d (Supporting Information) show survey spectra of MXene ink and MXene dispersed in water, respectively. The atomic percentage for the O 1s signal was measured at 28.1% and 45.6% for the co‐solvent based ink and MXene dispersed in water, respectively, confirming a higher oxidization level for MXene in water. This corresponds to an ≈6% increase in the oxidization level for the formulated ink and a substantial ≈23% increase for water‐dispersed MXenes, compared to freshly exfoliated MXene flakes. These results confirm the greater chemical stability of MXene nanosheets in the co‐solvent system. Furthermore, the core level scans of Ti 2p (Figure 2h,i) and C 1s peaks (Figure S3e, Supporting Information) demonstrate an increase in the amount of TiO_2_ and a nearly complete conversion of the MXene to TiO_2_ in water‐dispersed MXenes, further confirming the higher chemical stability of the formulated ink.

In addition to meeting the ink's physical and chemical stability requirements, the wettability of the ink‐substrate system was also assessed, as it plays a significant role in the formation of uniform films.^[^ ^63^ ^]^ The contact angle of the MXene ink was measured via the sessile drop method, yielding a value of θ = 30° on a 1 × 1 cm^2^ printed gold on polyimide over a recording time of 10 s (Figure S4a, Supporting Information). Moreover, the formulated ink was dropcast on various substrates, including silicon, polyimide, and glass, to assess potential coffee ring issues while drying (Figure S4b–d, Supporting Information). The optical images of the samples demonstrated a uniform film without any coffee ring effect after annealing at 250 °C in an inert atmosphere, confirming the proper co‐solvent system. The co‐solvent system effectively creates a surface tension gradient during drying, which helps to mitigate the coffee ring issue (Marangoni effect).^[^ ^64^ ^]^ Thermogravimetric analysis (TGA) was performed to determine the annealing temperature for the post‐printing process. The ink was loaded into an alumina crucible for measurements. The TGA results (Figure 2j) show a mass loss of ≈85% up to 180 °C due to the solvent evaporation, indicating that 200–250 °C is a suitable temperature to remove all solvents in post‐printing processes.^[^ ^65^ ^]^

### 2.3 Optimization of Aerosol Jet Printing

AJP relies on either an ultrasonic or a pneumatic atomizer to generate dense aerosolized droplets of the functional ink, which are then transported to the printhead using an inert carrier gas, typically nitrogen, and precisely deposited onto a substrate.^[^ ^38^, ^58^ ^]^ Atomization yield and droplet size distribution are influenced by the physical properties of the fluid, such as viscosity, surface tension, and density, as well as the instrument parameters, including atomization power and frequency for ultrasonic atomization.^[^ ^58^ ^]^ The inverse of the Ohnesorge number (Z), given by Z =$\sqrt{\gamma \rho D}/\eta \;$, where γ, ρ, D and η represent surface tension, density, nozzle diameter, and viscosity, respectively, measures droplet jetting behavior and predicts the stability of ink droplet generation. Direct comparisons between Reynolds and Ohnesorge numbers are not applicable to AJP, as droplet generation occurs in the ink vial subject to ultrasonic energy, while jet formation happens at the nozzle.^[^ ^66^ ^]^ In our previous work, we demonstrated that a commercial AJP utilizes an atomizer equipped with a transducer operating at a frequency of 1.67 MHz, resulting in droplets ranging from 1 to 5 µm in diameter.^[^ ^66^ ^]^ Optimization of the Z number for AJP, typically ranging from 2 to 6, consistently exhibits favorable jetting properties. Based on the rheology properties of the formulated MXene ink, the calculated Z number of 2.6 confirms the printability of the developed ink for AJP using an ultrasonic atomizer.

To evaluate the resolution of the aerosol jet printer with the formulated MXene ink and to achieve uniform deposition with minimum overspray, an array of lines was printed on a glass substrate using a 300 µm diameter nozzle at various focusing ratios (FR), defined as the sheath to carrier gas flow rate ratio.^[^ ^67^ ^]^ Additionally, other printing parameters, including water bath temperature, atomization current, and platen temperature, were optimized to enhance printing quality and kept constant during the experiment. The optimized printing parameters for MXene ink are summarized in Table S1 (Supporting Information). Figure S5a (Supporting Information) shows optical microscopic images of the printed lines on glass at various FR values varying from 0.5 to 2. The non‐uniform deposition was observed with an FR of 0.5, while high resolution and minimum overspray were achieved at FR values of 1 to 2. In contrast, when MXene nanoflakes dispersed in water were printed at similar FR values (Figure S5b, Supporting Information), low resolution and large overspray issues were encountered, confirming that the rheology properties of water are not suitable for AJP. Consequently, the ink composition has been optimized to achieve stable deposition over extended printing runs, with line arrays printed over 6 h showing great consistency and high‐resolution printing with a minimum overspray.

**Figure 3:**
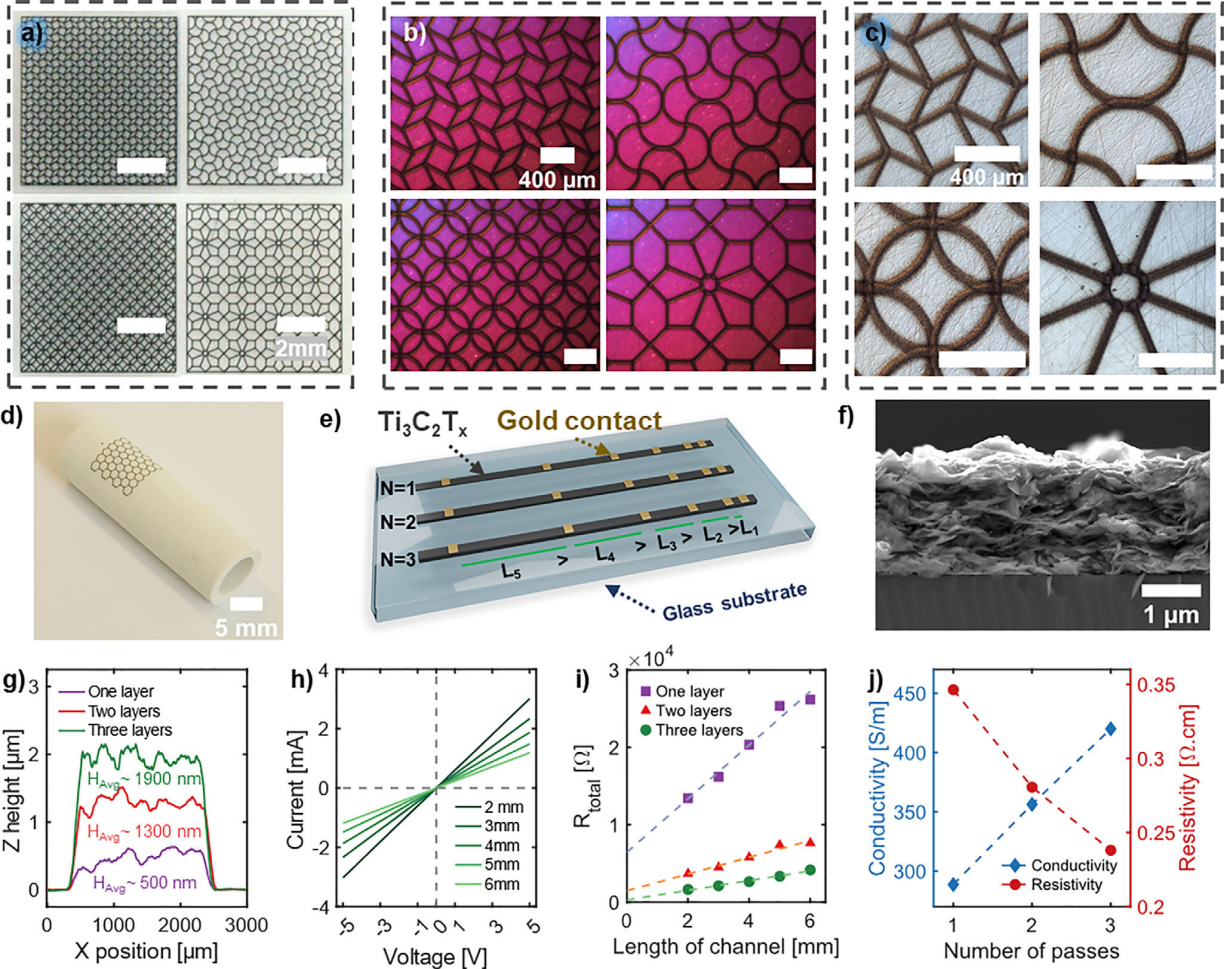
High‐resolution printing and electrical characterization of printed MXene. a) Optical image of printed complex structure on a sapphire substrate, demonstrating the high‐resolution printability of MXene ink and capability of AJP technology in printing small and complex features. b,c) High‐magnification microscopy images of printed complex structures on a sapphire substrate, corresponding to the structure shown in (a). d) Optical image of printed MXene ink on alumina tube with 1 cm inner diameter, highlighting the conformal printing capabilities of AJP technology. e) Schematic illustration of transmission line measurements (TLM) method on a glass substrate, showing varying numbers of printed passes (1, 2, and 3) of MXene ink and gold contact pad lengths of 2 mm, 3 mm, 4 mm, 5 mm, and 6 mm. f) Cross‐sectional SEM image of three printed passes on a silicon substrate. g) Profilometry analysis of one, two, and three passes printed MXene ink on a glass substrate, demonstrating the thickness variation. h) I‐V curve for three layers printed MXene ink. i) Resistance versus length of channel plot for the printed TLM structure. j) Conductivity and resistivity versus. The number of printed passes shows the effect of printed layers on electrical properties.

In addition, line widths of 66, 67, 68, 65, 59,57, 50, and 45 µm were achieved at various focusing ratios and printing speeds of 2, 4, 6 mm s^−1^ (Figure S6a–h, Supporting Information) using a 150 µm nozzle. More details can be found in Table S1 (Supporting Information). The line width versus focusing ratio (FR) plot (Figure S6i, Supporting Information) illustrates the combined effects of focusing ratio and printing speed on achieving high‐resolution printing. Notably, the highest resolution, with the line width of 45 µm, was achieved at FR of 2 and a speed of 6 mm s^−1^, demonstrating the high‐resolution printability of the developed ink and the capability of the aerosol jet printing for uniform deposition of the dispersed 2D material solutions. Using AJP, uniform MXene lines (minimum line widths of 45 µm) with precise line gaps ranging from 50 to 20 µm were successfully achieved (Figure S7a–e, Supporting Information). This highlights the adaptability of the AJP technique and compatibility of formulated MXene ink to depositing complex patterns with small feature sizes on a wide range of substrates, including both curved or planar surfaces. High‐resolution complex structures were successfully printed on sapphire and alumina tubes (**Figure** **3**a,d), highlighting the capability of AJP for both high‐resolution and conformal printing. Table S2 (Supporting Information) summarizes the width line and gap values reported from previous works.

### 2.4 Electrical Characterization of Aerosol Jet Printed MXene

The electrical properties of aerosol jet printed Ti_3_C_2_T*_x_* were determined using a transmission line measurements (TLM) structure with a varying number of printed layers and gold contact pads on a glass substrate (Figure 3e). Ti_3_C_2_T*_x_* lines (2 mm × 30 mm) were printed on a glass substrate with one, two, and three printed layers. Five different lengths were used for conductivity measurements (2, 3, 4, 5, and 6 mm) with 1 mm × 2 mm gold contacts printed on top of the MXene in a TLM structure. The printed MXenes were annealed under Ar gas at 250 °C for 2 h. Figure S8a,b (Supporting Information) shows a top‐down microscopic image and magnified SEM image of annealed printed MXene lines, demonstrating uniform deposition of MXene ink.

Cross‐sectional SEM imaging and stylus profilometry were conducted to analyze the height profile of the printed MXene lines with increasing the number of print passes (Figure 3f,g; Figure S9a–e, Supporting Information). The data confirmed a direct correlation between the number of passes and the increase in Z height, with the average thickness reaching 0.5, 1.3, and 1.9 µm for one, two, and three passes respectively. Cross‐sectional SEM images of printed MXene on the silicon substrate after sintering exhibited trends consistent with the profilometry measurements. Current‐voltage (I‐V) curves for one, two, and three passes at different gold contact lengths (Figure 3h; Figure S8c,d, Supporting Information) demonstrated ohmic characteristics, with an increasing number of passes reducing resistance. The resistance of each TLM structure versus channel length (Figure 3i) showed a linear relation. The specific values of conductivity and resistivity for our printed Ti_3_C_2_T*_x_* ink are 290 S m^−1^ and 0.35 Ω.cm for one pass, 360 S m^−1^ and 0.28 Ω.cm for two passes, and 420 S m^−1^ and 0.24 Ω.cm for three passes (Figure 3j).

Theoretical and experimental studies on porous materials suggest that capacitance performances depend on pore quantity, surface area, and active sites.^[^ ^68^ ^]^ Higher electrode microscale porosity increases the electrochemical reaction area and decreases the ion transport resistance, enhancing the electrochemical performance of electrode materials.^[^ ^69^ ^]^ Cross‐sectional SEM images of aerosol jet‐printed MXene nanoflake thin films revealed porous microstructures with nano‐and micro‐scale cavities formed by stacked Ti_3_C_2_T*_x_* layers (Figure S10a, Supporting Information). These diverse cavity systems can conceivably enhance the electrochemical performance of printed devices by providing abundant intercalation sites for given the intricate nature of the films. The SEM images exhibited a high degree of complexity and significant variability in lighting, rendering statistical pore analysis difficult (Figure S9, Supporting Information). Using the Sauvola method, the global microscale porosity of the image and therefore the printed thin film, was estimated to be 26.85%. More details can be found in the Supplementary Information section (Figures S10–S12, Supporting Information).

### 2.5 Electrochemical Performance of Printed MXene Electrodes

We explored the potential application of aerosol jet printing with our developed MXene ink as SCs. Figure 1 illustrates the schematic of the overall fabrication process of the Ti_3_C_2_T*_x_* MXene supercapacitor. Here, custom‐formulated gold nanoparticles were chosen as the current collector due to high electrical conductivity, low contact resistance with Ti_3_C_2_T*_x_*, strong adhesion to polyimide substrate after sintering, and excellent electrochemical stability.^[^ ^70^ ^]^ Following the printing, gold was sintered at 350 °C for 30 min. The current collector consists of an IDE pattern with four fingers on each side, the Ti_3_C_2_T*_x_* MXene ink was then printed on top of the current collector as an active layer and subsequently annealed at 250 °C for 2 h.

**Figure 4:**
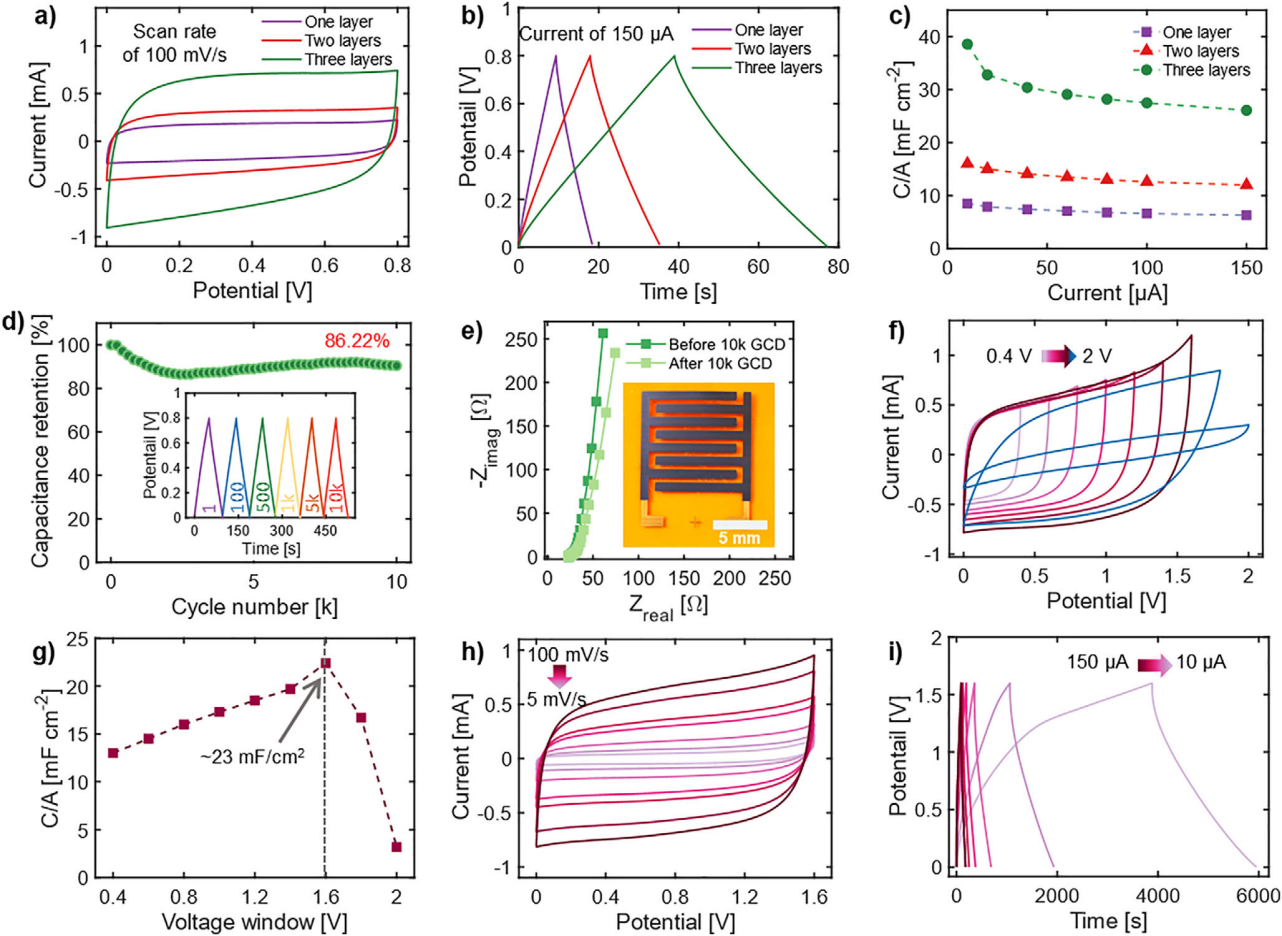
Electrochemical performance of printed Ti_3_C_2_T*_x_* supercapacitor in NaClO_4_/PC organic electrolyte. a) Comparison of cyclic voltammetry (CV) curves for one, two, and three printed layers at 100 mV s^−1^ scan rate. Three passes of MXene SCs show a larger CV area. b) Comparison of galvanostatic charge‐discharge (GCD) curves for one, two, and three printed layers at 150 µA current. c) Areal capacitance was calculated from GCD curves for one, two, and three three‐layer devices at different currents. d) Cycling stability of aerosol jet printed MXene SCs with three layers at 150 µA over10 000 cycles. The inset shows GCD curves at various cycles, demonstrating the capacitive behavior of the devices. e) Nyquist plots for the three‐layer MXene SC before and after 10 000 GCD cycles at 150 µA current. f) CV curves of a three‐layer MXene SC at different potential windows of 0.4, 0.6, 0.8, 1, 1.2, 1.4, 1.6, 1.8, and 2 V at a scan rate of 100 mV s^−1^, used to optimize the operating potential windows. g) Areal capacitance values are calculated from the CV curves at different voltage windows. h) CV curves at scan rates of 5, 10, 20, 40, 50, 80, and 100 mV s^−1^ for a potential window of 1.6 V. i) GCD curves at different currents of 10, 20, 40, 60, 80, 100, and 150 µA for a potential window of 1.6 V, illustrating the increase in performance of the SC by increasing the voltage window.

To correlate the active material loading with electrochemical performance, three distinct MXene devices were printed with one, two, and three printed passes with a total geometric area of 2.2 cm^2^ for printed MXene (shown in the inset is **Figure** **4**e). The MXene SCs devices were electrochemically characterized using cyclic voltammogram (CV), galvanostatic charge–discharge (GCD), and electrochemical impedance spectroscopy (EIS) using sodium perchlorate (NaClO_4_) solution in propylene carbonate (PC) (as an organic electrolyte). Rectangular cyclic voltammograms with no redox peaks (Figure S13a–c, Supporting Information) along with triangular GCD curves (Figure S13d–f, Supporting Information) indicate an electrical double‐layer capacitance behavior charge storage mechanism.^[^ ^35^ ^]^ The increase in the number of printed MXene layers leads to larger capacitance values (Figure 4a,b). The areal capacitances of 8.5, 16, and 39 mF cm^−2^ at a current of 10 µA were achieved for printed MXene SCs with one, two, and three printed passes, respectively (Figure 4c).

The Supporting Information contains details of the capacitance calculations, and Tables S3–S5 (Supporting Information) summarize the performance metrics for the devices, including areal capacitance, volumetric capacitance, energy density, and power density. The areal and volumetric capacitance of each device were calculated based on obtained CV and GCD tests (Figure S14a–c, Supporting Information). Three‐layer MXene SCs display good cycling stability with capacitance retention of ≈86.22% after 10000 continuous GCD cycles (Figure 4d). Figure 4e shows the Nyquist plot of a three‐layer device, obtained from EIS before and after 10000 GCD cycles. The Nyquist curve has a greater slope in the low‐frequency region before 10000 GCD cycles, indicating a significant involvement of the diffusion‐controlled component and a faster rate of ion penetration into the interlayer of Ti_3_C_2_T*_x_* MXene nanosheets.

A series of CV tests were performed at various voltages from 0.4–2 V to optimize the operating voltage window for organic‐based electrolytes (Figure 4f). The capacitance increases with increasing the operating voltage window from 0.4 to 1.6 V (e.g., 23 mF cm^−2^ at 1.6 V). However, a further increase in the voltage window beyond 1.6 V resulted in a decrease in capacitance because of the dissociation of PC (Figure 4g). The CV and GCD curves were performed at various scan rates and currents within the voltage window of 0–1.6 V (Figure 4h,i). The areal and volumetric capacitance of 47 mF cm^−2^ and 169 F cm^−3^ are calculated at a current of 10 µA, respectively, indicating the increase in the electrochemical performance of MXene SC with an increase in the operational voltage window.

The effectiveness of the gold current collector was validated by printing an MXene SC device with three printed passes without the gold current collector and subsequently analyzing the electrochemical performance of SC in an organic electrolyte (Figure S15a–c, Supporting Information). The conductivity of printed MXene electrodes was insufficient to efficiently deliver electrons to the external power sources, impacting the collected data from CV, GCD, and EIS. The EIS measurement (Figure S15c, Supporting Information) shows a large intrinsic resistance of ≈8 kΩ.

**Figure 5:**
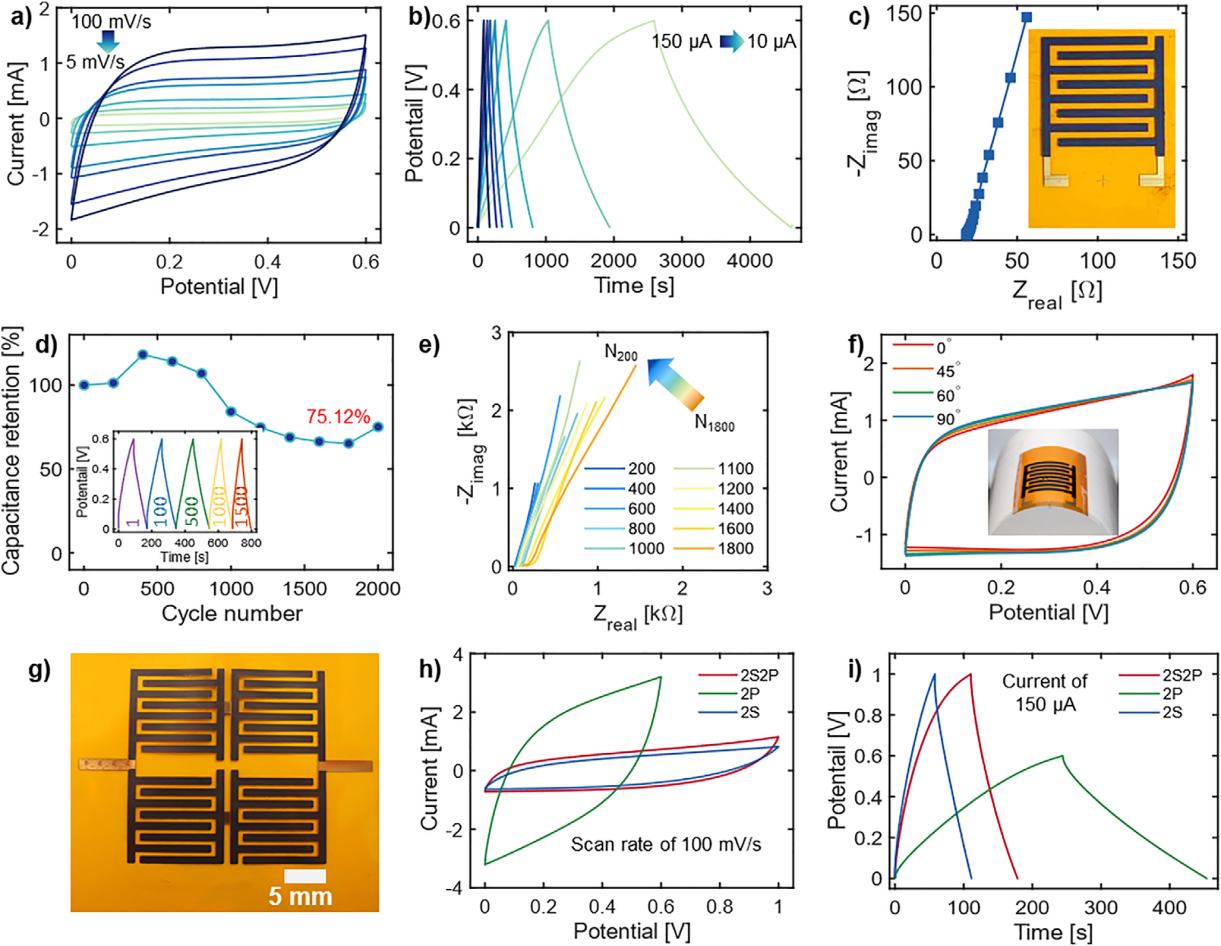
Electrochemical performance of three‐layer printed Ti_3_C_2_T*_x_* supercapacitor in aqueous H_2_SO_4_‐PVA gel electrolyte. a) CV curves were recorded at scan rates of 5, 10, 20, 40, 50, 80, and 100 mV s^−1^. b) GCD curves at various current values of 10, 20, 40, 60, 80, 100, and 150 µA. c) EIS results in an aqueous electrolyte. The inset shows an optical image of MXene SCs on a polyimide substrate. d) Cyclic stability of aerosol jet printed MXene SCs at 150 µA over 2000 cycles. The inset displays the GCD curves, highlighting the capacitive retention at different cycles. e) Nyquist plots were recorded after every 200 GCD cycles, from 200 to 1800 cycles, providing insights into the electrochemical performance in aqueous electrolytes. f) CV curves were recorded at various bending states of 0°, 45°, 60°, and 90°. The inset shows a photograph of MXene SCs on a polyimide substrate, bent at 60°. g) Photograph of a 2 × 2 array of MXene SC (configured as 2S2P) with three printed MXene layers. h) CV curves for various configurations, including two devices in series (2S), two devices in parallel (2P), and a 2 × 2 array of devices (2S2P), measured at a scan rate of 100 mV s^−1^. i) GCD curves corresponding to the same configurations (2S, 2P, and 2S2P) at a current of 150 µA.

The electrochemical performance of three‐layer printed MXene SCs was evaluated with the same geometric area (2.2 cm^2^, shown in the inset is **Figure** **5**c) using sulfuric acid (H_2_SO_4_)‐poly‐vinyl alcohol (PVA) aqueous gel electrolyte (Figure 5a,b). The areal and volumetric capacitances of 122 mF cm^−2^ and 611 F cm^−3^ (current of 10 µA) were calculated, respectively, which exceed previously reported values for printed MXene SCs. The higher capacitance obtained from the aqueous gel electrolyte compared to the organic electrolyte is due to the higher ion mobility of protons in the aqueous electrolyte. Moreover, in an organic electrolyte, the surface terminal groups on MXene sheets are electrochemically inactive, leading to lower capacitance.^[^ ^71^ ^]^ Figure 5c shows the EIS of MXene SCs using aqueous gel electrolyte, indicating the double‐layer capacitance behavior of printed MXene SCs, consistent with the CV and GCD results. The MXene SCs with the gel electrolyte demonstrated cycling stability with capacitance retention of ≈75.12% after 2000 continuous GCD cycles (Figure 5d).

To gain a deeper understanding of the cycling capacitance behavior of MXene SCs in aqueous gel electrolyte, EIS analysis was conducted after every 200 GCD cycles, from 1 to 2000 cycles, as shown in Figure 5e and Figure S16a (Supporting Information) (zoomed region of the high‐frequency region). The EIS analysis demonstrates the increase in electrode resistance, electrolyte resistance, and diffuse layer resistance with increasing the GCD cycles. In addition, an increase in capacitance was observed between 200–400 GCD cycles (Figure 5d). We hypothesize that this increase in capacitance can be attributed to two primary factors. First, the presence of water molecules in the electrolyte may lead to the formational additional functional groups, such as ─OH, on the MXene sheets, which could enhance capacitance. This effect can be correlated with the size of MXene particles, as smaller particles with more edges and defects are more reactive with water. This trend is consistent with previous studies on inkjet‐printed MXene SCs, where an initial capacitance increase was also reported.^[^ ^35^ ^]^ However, no such increase in capacitance was observed in extrusion printing of MXene SCs,^[^ ^35^ ^]^ where larger MXene sheets were employed, nor in our work when organic electrolytes were employed (Figure 4d). Second, the observed trend may be related to the gradual diffusion of hydrogen ions into the inner layer of MXene SCs over time. Given the high viscosity of the gel electrolyte, ion diffusion and complete coverage of all MXene sheets within the device may require additional time.

Despite the relatively lower cycling stability in aqueous gel electrolytes compared to organic‐based electrolytes, the higher areal and volumetric capacitance values highlight their potential of applications requiring higher capacitance. The printed MXene SCs on polyimide substrate exhibited excellent mechanical durability (Figure 5f), delivering a consistent CV response during the bending angle test. To demonstrate the practical application potential of MXene SCs across a wide voltage range, individual MXene SCs were integrated into two devices in series (2S), two devices in parallel (2P), and a 2S2P array of devices (Figure 5g) to meet the requirements for high voltage and capacitance. The Corresponding CV and GCD curves (Figure 5h,i) exhibit the expected results: the series configuration (2S) extended the operating voltage window to 1 V, while parallel connection doubled current output and discharge time. The 2S2P array exhibited excellent electrochemical performance, maintaining rectangular CV curves and triangular GCD curves within the extended 1 V window Figure S16b and Video S1 (Supporting Information) demonstrate the practical application of integrated 2S2P MXene SCs in powering a temperature‐humidity sensor, operated for ≈30 s.

**Figure 6:**
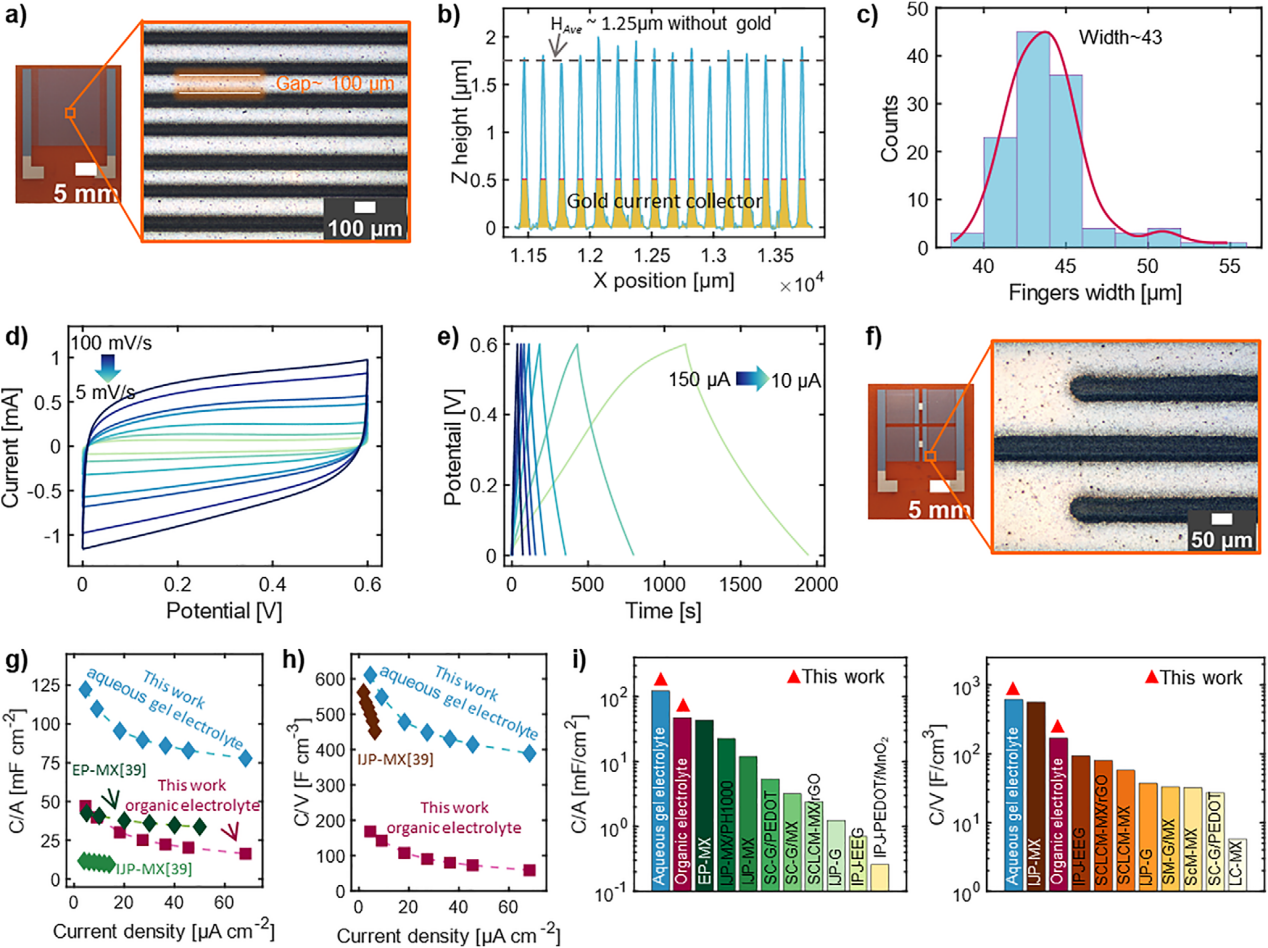
a) Optical image of a high‐resolution MXene (HR‐MXene) SC printed on a polyimide substrate. The magnified image of the selected area highlights uniform ink deposition and small printed features. b) Profilometry analysis along the SC fingers shown in (a). The gold/MXene SC has an average thickness of 1.75 µm, with the gold current collector at 0.5 µm and the MXene layer on top at an average thickness of 1.25 µm. The thickness profile of the gold current collector is shown in Figure S16 (Supporting Information). c) Variation in the line width of printed fingers, corresponding to (b). Line width measurements were taken at a thickness of 0.5 µm, excluding the gold layer. d) CV cures for HR‐MXene SCs at various scan rates. e) GCD curves for HR‐MXene SCs at various currents. f) Optical image of high‐resolution 22 arrays, with a magnified image of the selected area. g) Comparison of the areal capacitance of inkjet and extrusion printed MXene^39^ with this work in gel electrolyte, showing the superior performance of the aerosol jet‐printed MXene SCs. h) Comparison of the volumetric capacitance of this work with inkjet printing (IJP) MXene^39^ in gel electrolyte, confirming the higher *C*/*V* for aerosol jet printed MXene. i) Areal capacitance of aerosol jet printed MXene compared with previously reported SCs. j) Volumetric capacitance of aerosol jet printed compared with previously reported SCs. EP‐MX: extrusion‐printed MXene, IJP‐MP/PH1000: inkjet‐printed MXene/PH1000, IJP‐MX: inkjet‐printed MXene, IJP‐G: inkjet‐printed graphene, SM‐G/MX: spray‐masked graphene/MXene, SC‐G/MX: spray‐coated graphene/MXene, SC‐G/PEDOT: spray‐coated graphene/PEDOT, SCLCMX/rG: spray coated and laser cut mask MXene/reduced graphene, IJP‐EEG: inkjet‐printed electrochemical exfoliated graphene, G‐Q Dot: graphene quantum dot, SC‐G: Spray‐coated graphene, SCLCMX: spray coated and laser cut mask MXene, ScM‐MX: Scratch method MXene, Transparent‐MX: transparent MXene. Detailed references and specific values in (g)–(j) can be found in Tables S3–S5, Supporting Information.

To further explore the potential of AJP in fabricating high‐resolution devices, an MXene SC was printed on a polyimide substrate, and its electrochemical performance was evaluated (**Figure** **6**). Figure 6a illustrates an optical image of high‐resolution MXene (HR‐MXene) SCs (geometric area of 1.6 cm^2^) along with a magnified image of the zoomed section, highlighting minimal overspray. The stylus profilometry on high‐resolution MXene SC shows that the gold current collector has an average thickness of 0.5 µm (Figure 6b; Figure S17c, Supporting Information), while the deposited MXene active material thickness is ≈1.25 µm. In addition, the average line width of 43 µm was calculated using the profilometry data along the fingers (Figure 6c).

The CV and GCD curves corresponding to high‐resolution MXene (HR‐MXene) SCs (Figure 6d,e) demonstrate the electrical double layer charge storage mechanism, with the calculated areal and volumetric capacitance of 67 mF cm^−2^ and 535 F cm^−3^ (current of 10 µA), placing them among the highest‐performing printed supercapacitors reported to date and highlighting the exceptional capability of AJP technique in fabrication of electronic and energy storage devices. Figure 6f shows an optical image of the 2S2P array of high‐resolution MXene SCs, accompanied by a magnified image of the selected area, confirming the high‐resolution printed lines and uniform deposition of developed MXene ink achieved using the AJP technique, demonstrating the scalability of our printing process. Details on the profilometry analyses, CV, and GCD curves are provided in Figure S17a–f (Supporting Information).

The areal and volumetric capacitance of aerosol jet‐printed MXene SCs in organic and gel electrolytes were compared to previous SCs (Figure 6g–j; Tables S3 and S4, Supporting Information), indicating higher values than previously reported printed SCs. Aqueous gel electrolytes showed higher areal and volumetric capacitances than reported inkjet printers and extrusion‐printed MXenes.^[^ ^35^ ^]^ The areal energy density and power density of aerosol jet‐printed MXene and other 2D SCs were compared using a Ragone plot (Figure S18, Supporting Information). The calculated areal energy densities and power densities for MXene SCs in organic electrolytes reach as high as 16.77 µWh cm^−2^ and 29.12 µW cm^−2^, respectively. In addition, areal energy densities and power densities for MXene SCs in aqueous gel electrolyte are calculated as 6.11 µWh cm^−2^ and 10.92 µW cm^−2^, respectively (Figure 5c). The higher areal energy and power densities for organic‐based electrolyte correlate to the broader voltage window (e.g., 1.6 V for organic electrolyte), as energy density is directly proportional to the square of the cell voltage. The achieved energy densities are higher than previously reported SCs, including, inkjet‐printed Ti_3_C_2_T*_x_*/PH1000^[^ ^72^ ^]^ (0.28 µWh cm^−2^), extrusion printed Ti_3_C_2_T*_x_* ^[^ ^35^ ^]^ (0.32 µWh cm^−2^), and Ti_3_C_2_T*_x_*/rG^[^ ^73^ ^]^ (0.26 µWh cm^−2^), showing a potential of aerosol jet‐printed‐MXene inks for developing flexible micro‐supercapacitors for IoT and small electronic devices.

## 3 Conclusion

In this work, we developed a chemically and physically stable, high‐performance Ti_3_C_2_T*_x_* MXene ink suitable for aerosol jet printing (AJP), offering a significant advancement in the fabrication of printed energy storage devices. Comprehensive ink characterization was conducted to optimize ink stability and printability, confirming the compatibility of our formulated ink with AJP through consistent printing behavior and minimal overspray observed over an extended 6 h period. The calculated sheet resistance demonstrated that increasing the number of passes decreases resistivity, and the printed Ti_3_C_2_T*_x_* with *n* = 3 has the lowest resistivity of 0.24 Ω.cm. We estimated the porosity of printed MXene ink on silicon using an SEM cross‐section, revealing a global porosity of 26.85% for the printed MXene thin film. The electrochemical performance of the printed Ti_3_C_2_T*_x_* ink was studied using CV, GCD, and EIS tests, revealing a direct correlation between the thickness of the printed device and electrochemical performance in organic electrolytes. High‐resolution printing was achieved using AJP and the printed supercapacitor showed excellent electrochemical performance, including an areal capacitance of 67 mF cm^−2^ and a volumetric capacitance of 535 F cm^−3^ in an aqueous gel electrolyte. In addition, the printed supercapacitor showed excellent areal energy density of 16.77 µWh cm^−2^ and areal power density of 29.12 µW cm^−2^ in the organic‐based electrolyte, exceeding previous aerosol jet printed and inkjet printed MXene supercapacitors. The printed SC showed excellent cycling stability with capacitance retention of ≈86.22% after 10,000 continuous GCD cycles in the organic electrolyte. The development of printable MXene ink for aerosol jet printing holds promise beyond energy storage applications, offering potential in small electronics and sensors by leveraging the advantage of both MXenes and additive manufacturing techniques.

## 4 Experimental Section

### Ti_3_AlC_2_ MAX Phase Synthesis

Synthesis of Ti_3_AlC_2_ MAX phase was adapted from the work by Shekhirev et al.^[^ ^74^ ^]^ In this process, TiC, Ti, and Al powder were mixed and ball milled in a mass ratio of 2:1:1: for 18 h using zirconia balls. The ball‐milled mixture was annealed at 1400 °C for 2 h under an Ar atmosphere. The resultant material was powdered in a ball mill using zirconia balls and treated with 9 M HCl for 18 h to eliminate the impurities. Finally, the resulting powder was washed with water, vacuum‐dried at 80 °C for 6 h, and sieved using 400 mesh sieves to obtain MAX particles with a size smaller than 38 µm.

### Etching Ti_3_AlC_2_ MAX Phase

In this study, the minimally intensive delamination (MILD) synthesis method was employed to etch the Ti_3_AlC_2_ MAX phase and synthesize multilayered (*ml*)‐Ti_3_C_2_T*_x_* MXene, targeting larger flakes with fewer defects.^[^ ^48^ ^]^ Initially, the etchant solution was prepared by mixing 20 mL of 9 M hydrochloric acid (36.5 to 38.0%, Fisher Chemical) and 3.2 g lithium fluoride (98.5%, −325 mesh powder, Alfa Aesar) using a Teflon‐coated magnetic bar in a high‐density polyethylene (HDPE) bottle. Next, the solution was stirred for 5 min at 300 rpm to generate hydrofluoric (HF) acid in situ. Subsequently, 2 g of Ti_3_AlC_2_ MAX phase powder was gradually added to an acidic solution over 10 min to minimize the excessive bubbling that occurred due to the exothermic nature of this reaction. Following the complete addition of the MAX phase powder, the etching reaction was maintained under magnetic stirring at 500 rpm and 50 °C for 72 h. Isolation of the reacted material was achieved by diluting the acidic solution with ultrapure water and then by performing centrifugation at 3500 rpm for 5 min. The isolated material was then washed several times until the pH of the supernatant reached ≈5–6. Shaking was performed before centrifugation in each washing step to facilitate the intercalation of lithium ions and water molecules between delaminated sheets and to aid in the separation of black (*ml*)‐Ti_3_C_2_T*_x_* slurry from nonetched Ti_3_AlC_2_. At this point, the (*ml*)‐Ti_3_C_2_T*_x_* sediment started to swell and a distinctive black layer started to form on top of a grayish layer of mixed non‐etched Ti_3_AlC_2_/Ti_3_C_2_. The black (*ml*)‐Ti_3_C_2_T*_x_* slurry was carefully collected with a spatula and dried under vacuum at room temperature for 24 h.

### Liquid Phase Exfoliation (LPE) of (ml)‐Ti_3_C_2_T_x_ MXene

Solvent‐assisted exfoliation was employed using 30 mL of NMP as an organic solvent to reduce the size of the resultant multilayered (*ml*)‐Ti_3_C_2_T*_x_* MXene powder (≈200 mg) and produce nanosheets of Ti_3_C_2_T*_x_*. The solution was sonicated (Qsonica Q700) for ≈6 h at a power of 420 W (60% amplitude). Centrifugation was then performed on the resulting suspension at 4500 rpm for 5 mins to remove the unexfoliated and large flakes. Following centrifugation, the upper 80% of the centrifuged solutions were collected for characterization and ink formulation.

### Ti_3_C_2_T_x_ MXene Ink Formulation

The NMP‐based dispersion of few‐layered or single‐layer Ti_3_C_2_T*_x_* was prepared. The supernatant was collected after centrifugation, and twice the amount of 0.01 M NaCl solution (≈70 mL) was added to the dispersion.  The solution was centrifuged for 30 min at 21000 rpm to facilitate the flocculation of single or few‐layer Ti_3_C_2_T*_x_* flakes from the NMP. Then, the resulting Ti_3_C_2_T*_x_* flakes were washed with ethanol three times and dried under vacuum at room temperature for 6 h. Subsequently, 125 mg of isolated 2D‐layered Ti_3_C_2_T_x_ nanosheets were dispersed in a 5 mL mixture of water, ethanol, and ethylene glycol with a volume ratio of 5:4:1. Uniform dispersion of the ink was achieved after shear mixing at 7,000 rpm for 30 min using a Silverson L5MA Laboratory Mixer.

### Optimization of Printing Parameters for Ti_3_C_2_T_x_ MXene Ink

The Ti_3_C_2_T*_x_* electrodes and gold current collectors were fabricated using an Optomec Aerosol Jet printer (AJP) equipped with an ultrasonic atomizer and a 300 µm nozzle for MXene and gold NPs inks. Optimization of the AJP process parameters was performed to enable consistent and repeatable fabrications. First, PVP‐gold nanoparticles were synthesized based on our previous work.^[^ ^66^ ^]^ The resulting PVP‐AuNPs were then formulated into a 10–20 wt% PVP‐AuNP ink containing water, ethanol, and ethylene glycol as solvents. Subsequently, a polyimide substrate was placed on the platen stage of the printer and heated to 55 °C to facilitate solvent evaporation during printing. To achieve the desired thickness, three layers of gold ink were printed onto polyimide, which serves as a current collector layer. The printed gold structures were annealed at 350 °C for 30 min in an open‐air environment to obtain an end‐to‐end resistance of 4 Ω. Second, a functional layer was manufactured on the fabricated gold structure by printing a 25 mg mL^−1^ Ti_3_C_2_T*_x_* MXene nanoflakes while the platen was held at 50 °C. Three separate interdigitated patterns were manufactured for application as supercapacitors, distinguished by a varied number of printed passes (1 to 3). The printed supercapacitors were annealed for 2 h at 250 °C under an Ar atmosphere. Table S1 (Supporting Information) summarizes the details of aerosol jet printing parameters for both the gold and MXene inks.

### Material Characterization

The structure analysis and the surface chemistry of MXene were studied using X‐ray diffraction (XRD) and X‐ray photoelectron spectroscopy (XPS), respectively. The morphology study of the MXene, MAX phase, and printed MXene ink was performed using scanning electron microscopy. The statistical analysis of the size and thickness of exfoliated MXene was performed using atomic force microscopy. The rheological properties of formulated MXene ink were studied using a tensiometer and rheometer. The electrical conductivity of printed TLM structures was evaluated using a two‐point probe technique. A detailed description of characterization can be found in Supporting Information.

### Electrochemical Performance

The electrochemical performance of printed supercapacitors, including cyclic voltammetry (CV) and galvanostatic charge–discharge (GCD), was conducted using an SP‐50 BioLogic potentiostat. A two‐electrode system was employed with organic and aqueous electrolytes at room temperature. 1 M NaClO_4_/PC (organic) electrolyte was prepared by mixing propylene carbonate (PC, 99.5%) as the solvent and sodium perchlorate (NaClO_4_, 98%) as the ion‐donating salt. In addition, a 3 M H_2_SO_4_/PVA gel (aqueous) electrolyte was prepared using sulfuric acid (H_2_SO_4_, 97%) and PVA.^[^ ^75^ ^]^ The devices were tested at various CV scan rates of 5, 10, 20, 40, 50, 80, and 100 mV s^−1^ and GCD currents of 10, 20,40, 60, 80, and 100 µA in various voltage windows of 0.4–2 V for organic electrolyte and 0–0.5 V for aqueous electrolyte. The cyclic performance of the device with three passes was evaluated at 100 µA for 10,000 cycles. The detailed formulas for calculating electrochemical performance can be found in the Supporting Note.

## Conflict of Interest

The authors declare no conflict of interest.

## Author Contributions

F.R.K., D.E., J.E.K., M.S., and T.V.V. conceived the project. F.R.K., N.E.M., S.N., and H.B. synthesized the material. F.R.K. and T.V.V. Formulated the MXene ink. T.V.V. and J.E. synthesized and formulated gold ink. F.R.K. performed the rheology tests and contact angle study. F.R.K. and T.V.V. performed the aerosol jet printing. H.B. and K.C. performed scanning electron microscopy. N.M. performed a porosity study. F.R.K. measured the conductivity and data analysis. F.R.K. and N.E.M. performed electrochemical characterizations. F.R.K. and M.S. analyzed electrochemical data. F.R.K. wrote the manuscript with contributions from all co‐authors, all authors discussed the results and commented on the manuscript. C.E.S. and Y.G. guided the MXene development and participated in the discussion of the results and manuscript editing.

## Supporting information



Supporting Information

Supplemental Video 1

## Data Availability

The data that support the findings of this study are available from the corresponding author upon reasonable request.
