# Supplementary material for: StableTi3C2T x MXene Ink Formulation and High‐Resolution Aerosol Jet Printing for High‐Performance MXene Supercapacitors
Source: Small Methods. 2025 May 28;9(11):2500499. doi: 10.1002/smtd.202500499 (PMC12641374; doi:10.1002/smtd.202500499)
Supplement: Supplementary file 1 — Supporting Information [file SMTD-9-2500499-s001.docx]

**Supplementary Information**

**Stable Ti_3_C_2_T*_x_* MXene Ink Formulation and High-Resolution Aerosol Jet Printing for
High-Performance MXene Supercapacitors**

Fereshteh Rajabi Kouchi^1^, Tony Valayil Varghese^1^, Hailey Burgoyne^1^, Naqsh E Mansoor^1^, Myeong-Lok Seol^2^, Nicholas McKibben^1^, Shruti Nirantar^1-3^, Karthik Chinnathambi^1^, Josh Eixenberger^4,5^, Olivia Maryon^1^, Christopher E Shuck^6,7^, Yury Gogotsi^6^, Jessica E. Koehne^2^, David Estrada^1,5,8,*^

^1^Micron School of Materials Science and Engineering, Boise State University, Boise, ID 83725, USA

^2^NASA Ames Research Center, Universities Space Research Association, Moffett Field, CA 94035, USA

^3^School of Engineering, RMIT University, Melbourne, VIC, 3001 Australia

^4^Departmentof Physics, Boise State University, Boise, ID 83725, USA

^5^Center for Advanced Energy Studies, Boise State University, Boise, ID 83725, USA

^6^A.J. Drexel Nanomaterials Institute and Department of Materials Science and Engineering, Drexel University, Philadelphia, PA 19104, USA

^7^Department of Chemistry and Chemical Biology, Rutgers University, Piscataway, NJ 08854, USA

^8^Idaho National Laboratory, Idaho Falls, ID 83415, USA

*daveestrada@boisestate.edu

**Supplementary Figures**

**Supplementary Figure S1.** Material characterization: **a)** X-ray diffraction (XRD) pattern of Ti_3_C_2_T_x_ MXene and Ti_3_AlC_2_ MAX phase. **b)** SEM image of Ti_3_AlC_2_, indicating layered structure of precursor MAX phase. **c)** SEM image of (ml)-Ti_3_C_2_T_x_ MXene, revealing the expansion of layered structure due to the removal of Al layer of corresponded MAX phase. **d)** A survey XPS spectrum of (ml)-MXene. **e)** High-resolution XPS spectrum of Ti 2p for (ml)-Ti_3_C_2_T_x_ MXene. **f)** High-resolution XPS spectrum of C 1s for (ml)-MXene.


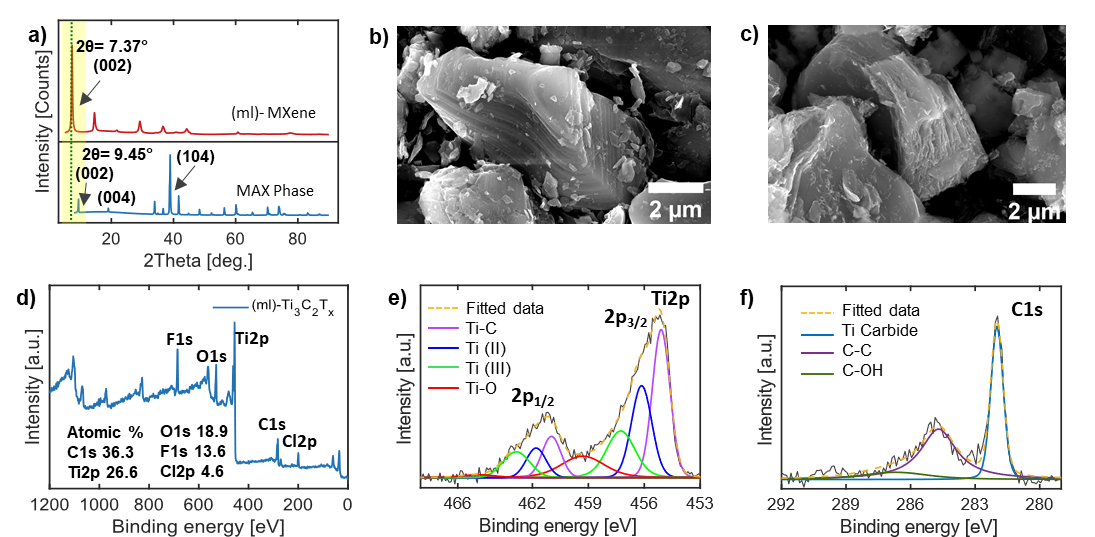


**Supplementary Figure S2.** **a)** AFM image of exfoliated MXene. **b)** Height profile along the line highlighted in Supplementary Figure S2a, showing thickness and roughness of the nanoflakes along the lines. **c)** AFM image of exfoliated MXene with larger scan size. Histogram of **d)** thickness and **e)** width of MXene nanoflakes, a total of 100 nanosheets were considered. **f)** A survey XPS spectrum of exfoliated MXene. There is a nitrogen peak (1.8 %) in the exfoliated MXene, which comes from the residual NMP solvent. **g)** High-resolution XPS spectrum of C 1s for exfoliated MXene.


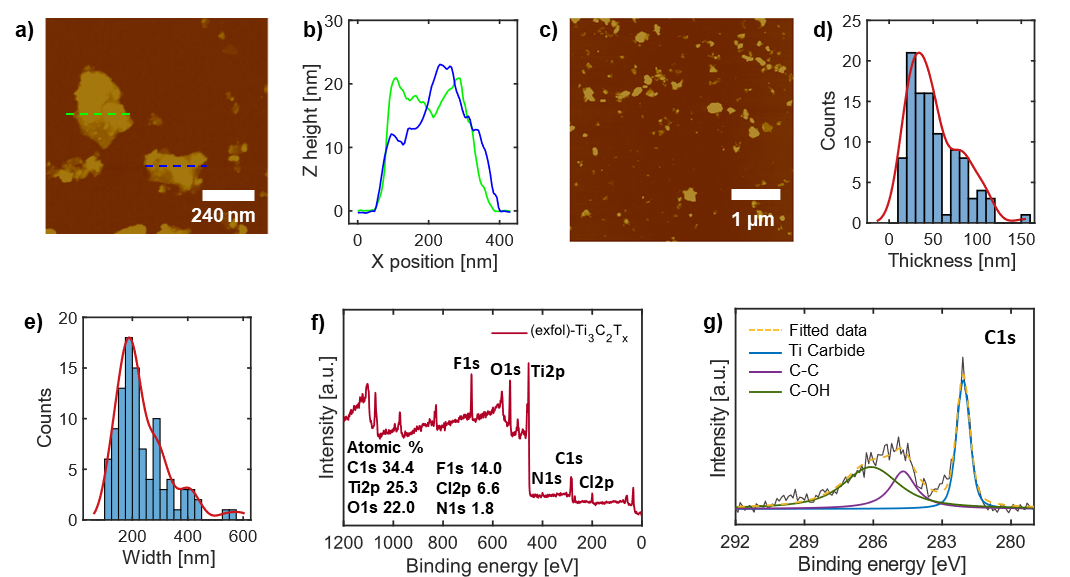


The nanoflake size is less than 400 nm, and the thickness is less than 15 nm. The lateral size of the nanosheets is one of the key parameters in aerosol jet printing. Large particles cause clogging in the printer nozzle. Therefore, the material size should be 1/50 of the nozzle diameter or even lower. In this study, the nozzle diameter is 300 µm, and the particle size was calculated to be within the favorable range for aerosol jet printing. The deconvoluted C 1s core-level of the exfoliated MXene shows a negligible increase in the intensity of the C-OH and C-O-C peaks, indicating the increase of C-OH, and C=O of functional groups on the surface of nanoflakes after exfoliation and washing step.

**Supplementary Figure S3.** **a)** Stability study of the formulated MXene ink (water: ethanol: ethylene glycol) over four months (concentration of 5 mg mL^-1^). The dispersed MXene nanoflakes are stable for three months. **b)** Optical images for stability study of the dispersed MXene in water with low concentration. **c**, **d)** Survey scan spectrum of MXene ink and dispersed MXene, respectively. **e)** The deconvoluted C 1s core-level spectrum of MXene.


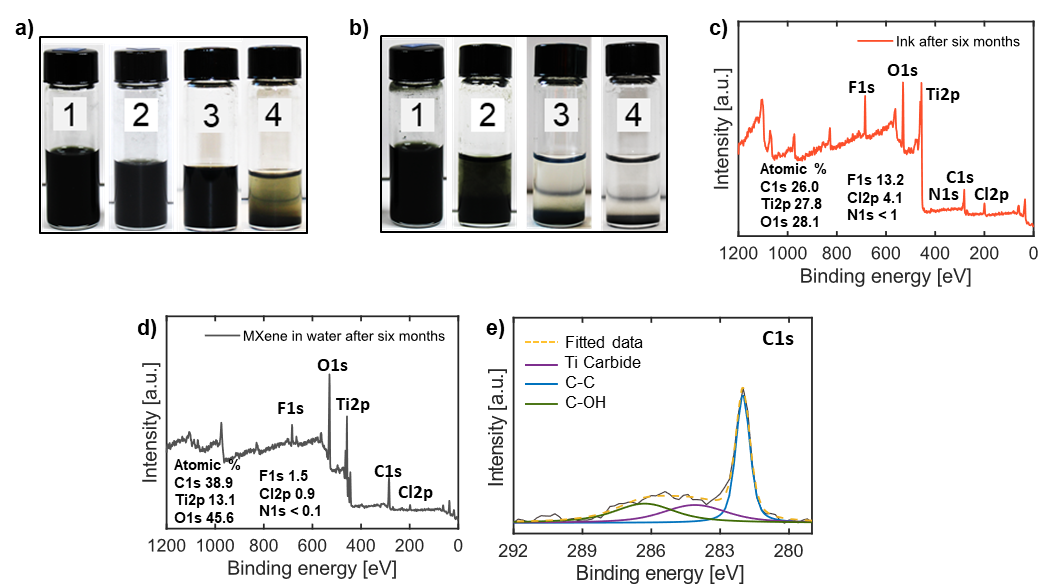

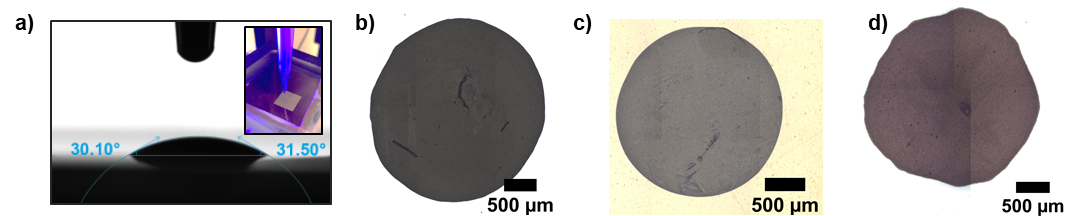


**Supplementary Figure S4. a)** Contact angle study of the formulated Ti_3_C_2_T_x_ ink on printed gold. Optical images of dropped cast ink on various substrates **b)** silicon, **c)** Kapton, **d)** glass. None of the substrates show a “coffee ring” effect, indicating the co-solvent system enables uniform drying of MXene films.


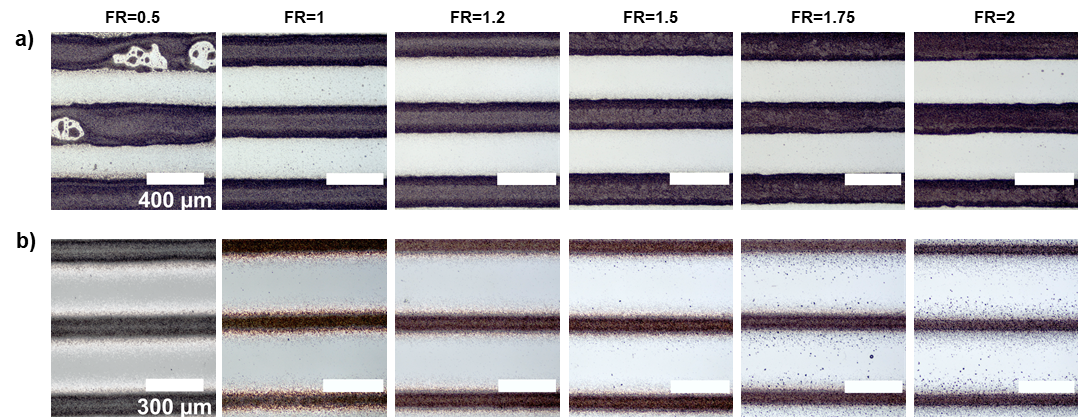


**Supplementary Figure S5.** Optimization of focusing ratio for **a)** the formulated ink and **b)** dispersed MXene in water. The carrier gas flow rate kept constant, while the sheath gas flow rate was changed to obtain various focusing ratio. The 300 µm nozzle was used here. Detailed data can be found in Supplementary Table S2.


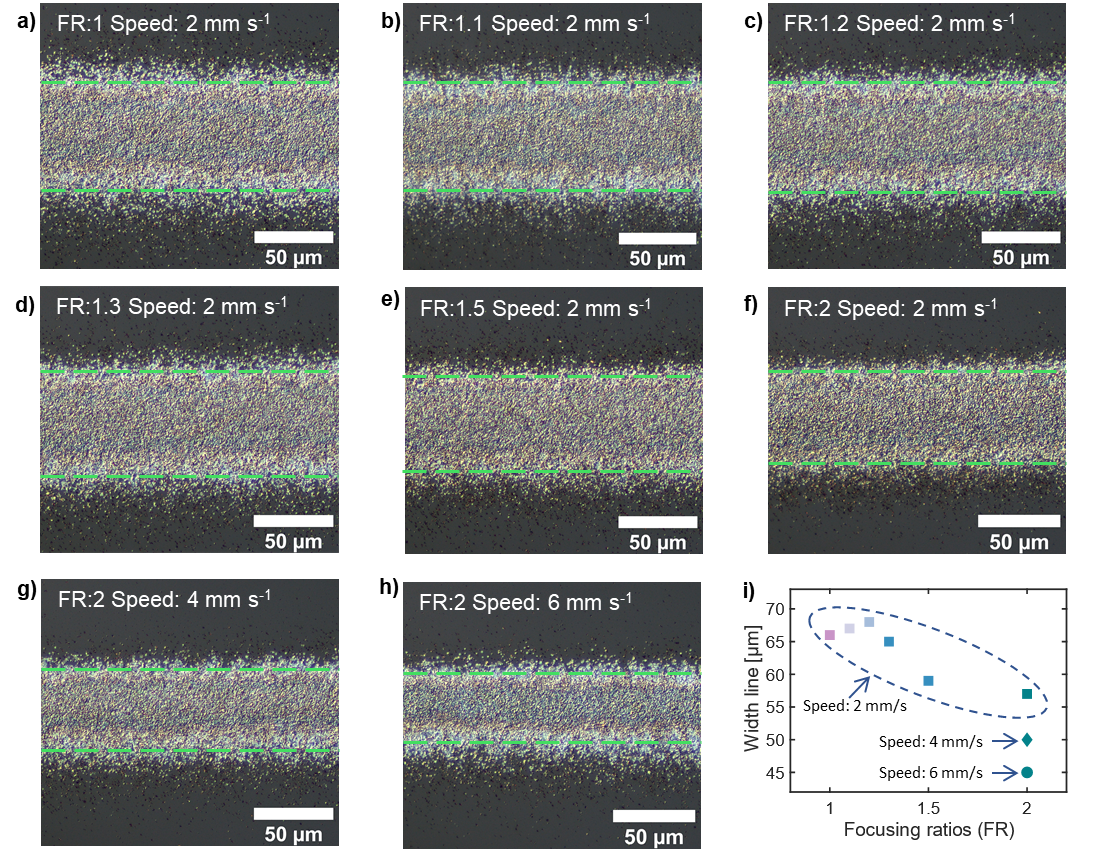


**Supplementary Figure S6.** Optimization of the focusing ratio using a 150 µm nozzle size to achieve high-resolution lines at different printing speeds of: **(a-f)** 2 mm s^-1^ (square) **(g)** 4 mm s^-1^ (diamond) **(h)** 6 mm s^-1^ (circle). While maintaining a constant carrier gas flow rate, the sheath gas flow was adjusted to achieve various focusing ratios (FR) in the range of 1-2. The resulting line widths for images (a-h) are 66, 67, 68, 65, 59, 57, 50, and 45 µm, respectively. **(i)** Line width vs. focusing ratio (FR) plot, showing the combined effects of focusing ratio and printing speed on achieving high-resolution printing. Square, diamond and circle correspond to speed of 2, 4, and 6 mm s^-1^, respectively. Detailed data can be found in Supplementary Table S2.


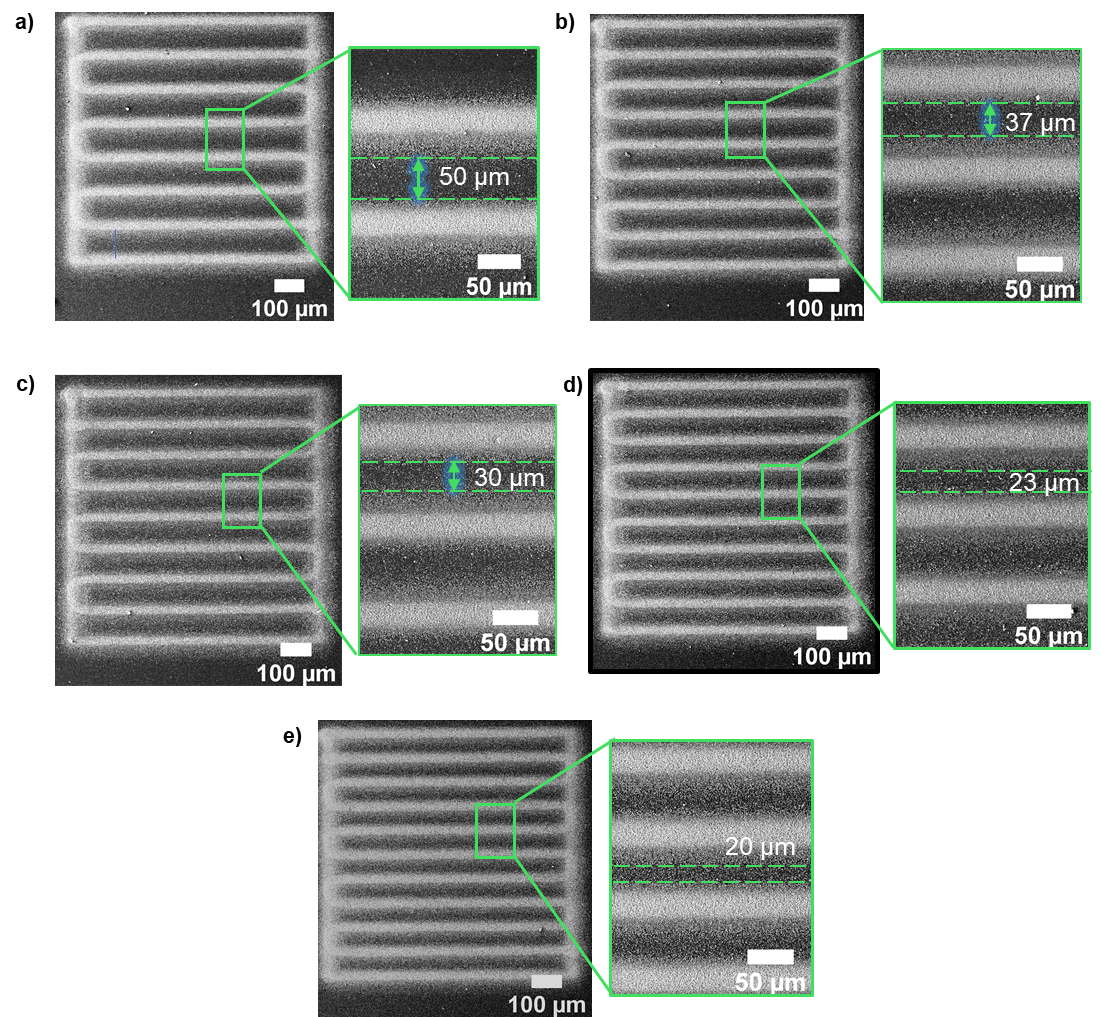


**Supplementary Figure S7.** SEM images studying the spacing between two printed MXene lines. The gaps between the lines are **(a)** 50 µm, **(b)** 37 µm, **(c)** 30 µm, **(d)** 23 µm, and **(e)** 20 µm.

Supplementary Figure S8a was obtained from optical microscopy (Carl Zeiss, Germany) using various magnifications after annealing at 30 °C for 2 h. It indicates the high-resolution and uniform aerosol jet printing of formulated MXene ink. The I-V curves for one, two, and three printed passes show the ohmic behavior.


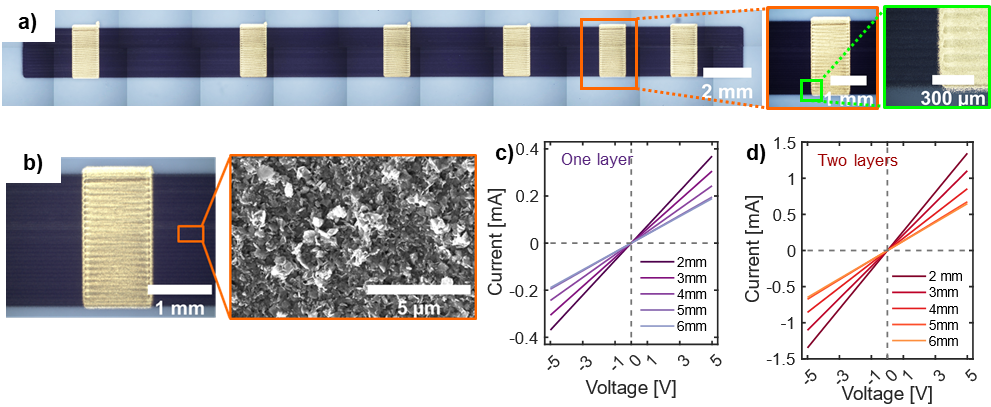


**Supplementary Figure S8.** **a)** Stitched microscopy of TLM structure with three passes of MXene ink. High resolution of printed MXene confirms the compatibility of formulated MXene ink with aerosol jet printing and well-optimized printing parameters. **b)** A microscopic image and a magnified SEM image of the printed Ti_3_C_2_T*_x_* line after annealing, demonstrating uniform AJP of MXene. **c-d)** I-V curves for one and two layers printed MXene.

**Supplementary Figure S9.** SEM images of cross-section of printed Ti_3_C_2_T*_x_* MXenes on silicon with **a-c)** for one, two, and three number of printed passes, respectively. The high-magnification SEM images of cross-sections for a single pass (**d**) and two passes (**e**).


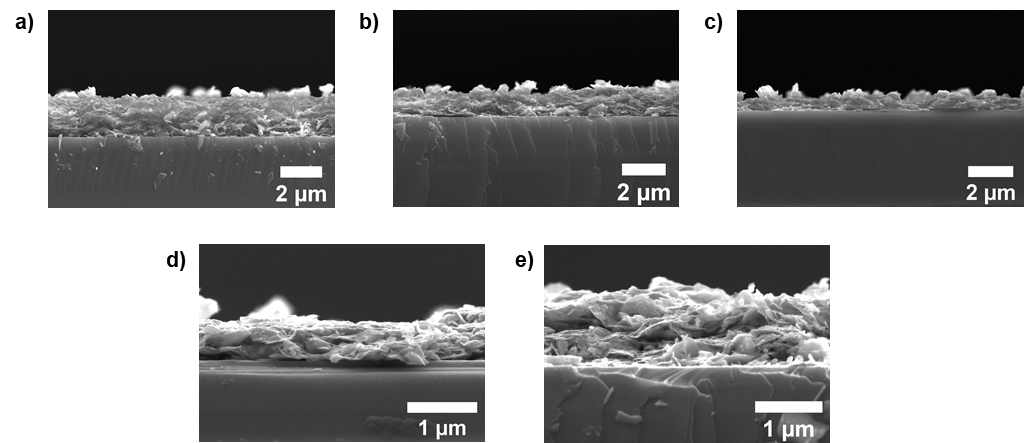


Global thresholding methods often overestimated porosity due to mono-modal histograms and exposure inconsistencies (Supplementary Figure S10b). To address these challenges, we employed the adaptive thresholding technique known as the Sauvola method^1^ (Supplementary Figure S10c, 11a-d,12a,b). A simple algorithm was developed to produce high-fidelity masks for the SEM images, which enhanced visualization, revealing complicated details when overlaid with the original image (Supplementary Figure S10d).


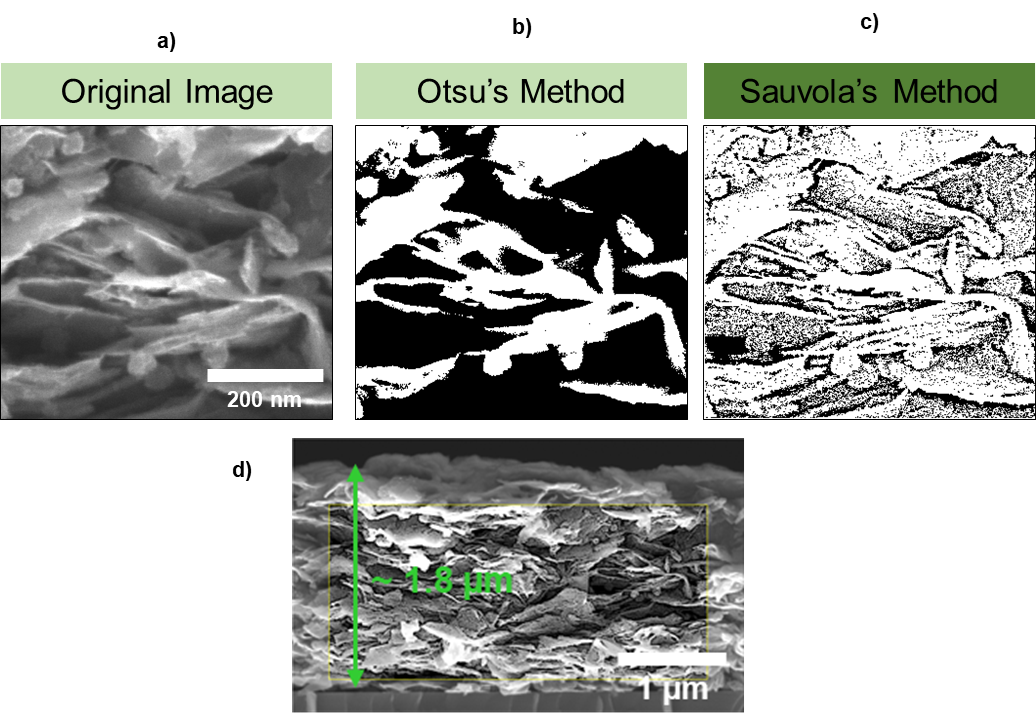


**Supplementary Figure S10.** SEM images of **a)** original image, **b)** Otsu’s method, **c)** Sauvola’s method. **d)** Cross-section SEM image of printed MXene ink with Sauvola Threshold Overlay. Three passes were deposited on a silicon substrate with a thickness of 1.8 µm, revealing the horizontal alignment of MXene nanoflakes stacked on top of each other.

In the Sauvola method technique, a local threshold (*T*) is calculated for each pixel based on the intensities of the surrounding neighborhood of pixels (*N*). The threshold is calculated as:

$$T_{N}=m_{N}\left[ 1+k\left( \frac{\delta_{N}}{R}-1 \right) \right]$$

where $m_{N}$ is the mean intensity value, which is calculated locally for a pixel from its surrounding neighborhood. $k$ is a user-determined sensitivity bias, $\delta_{N}$ is the standard deviation of the local neighborhood, and *R* is the threshold constant, which is traditionally fixed at 128 for grayscale images and is used as a cutoff for assigning binary pixel polarity.

It is typical to adjust the generated threshold mask by tuning the user-defined parameters *N* and *k*, but the best selection criteria for these parameters was originally unclear. Increasing *N* in the algorithm resulted in a smoother and more segmented binary mask, however, a total loss of fine detail was observed at higher *N* values (Supplementary Figure S11a). For neighborhood size, we found it prudent to keep a relatively low *N* value to preserve the fine detail captured within the SEM image. One downside to very low *N* values is that significant amounts of small noise are created within the output, observed as speckling or granulation, which served to convolute the statistical analysis of the mask.

**Supplementary Figure S11.** Sauvola method **a)** increasing neighborhood size (left to right). **b)** Pores count versus neighborhood size. **c)** pores count versus sensitivity**. d)** Adaptive vs. global thresholding techniques. **e)** Increasing sensitivity.


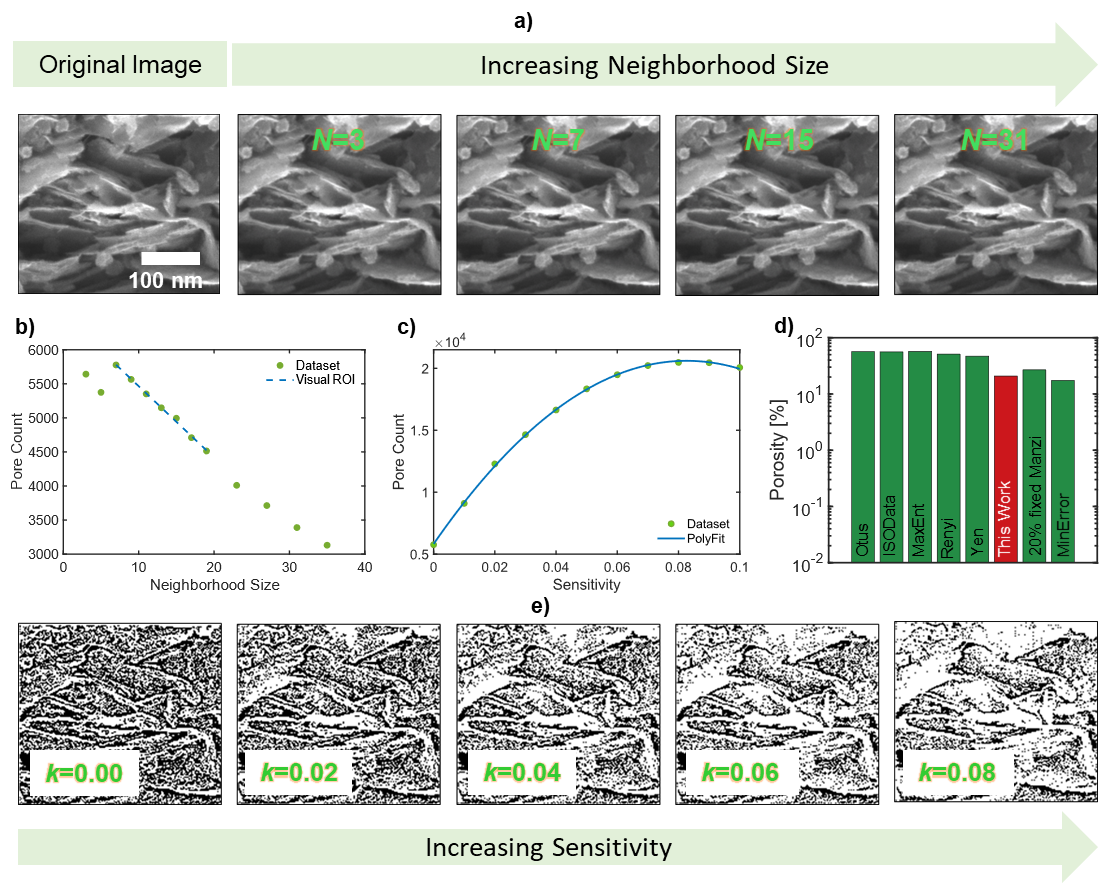


Therefore, to maximize mask resolution, we first visually established a region of interest (ROI) for *N* values that effectively struck a balance between small and large *N* values. The subjectivity of this approach was not satisfactory, though, particularly with respect to accuracy in data extraction and metric reproducibility, so we investigated relationships between *N* and the image properties of the output masks to establish an automated selection method for *N*. Based on a parameter sweep at *k*=0), a neighborhood size of *N*= 7 was selected for our image (Supplementary Figure S11b). This value was determined to be mathematically associated with the maximum number of pores for any neighborhood size, which we found statistically significant based on our previously mentioned parameter selection criteria. We found that the most effective binary masks were generated from an equalized image, and the optimal visual results were obtained by overlaying an equalized mask onto an image without equalization. This is because the contrast enhancement from equalization is intensified when applying an equalized mask onto an equalized image.

We selected our sensitivity value based on a second parameter sweep, which swept sensitivity values at *N*= 7, and we found a maximum value for pore counts at a sensitivity value of *k* = 0.08 (Supplementary Figure S11c). Increasing the *k* value of the algorithm causes the output image to become more saturated with white pixels (Supplementary Figure S11e), making the algorithm more sensitive to local variations within the image. Visual inspection was performed to verify that our selected mask suitably reduced noise while maintaining the key pore information from the image. The global porosity measurement obtained from this technique was benchmarked against some common literary approaches for estimating porosity from an image (Supplementary Figure S11d). The Sauvola method shows there is strong value in adaptive thresholding techniques for pore-segmentation and data extraction, particularly for images with complex mono-modal histograms, which tend to be overestimated by automatic global thresholding techniques. This approach provides accurate, local statistical pore analysis for materials and print optimization, enabling the development of high-performance energy storage devices with improved capacitive and electrical properties.

Prior to statistical analysis, the global porosity of the reconstructed images was calculated using the default global thresholding method in FIJI, and the threshold constant was adjusted to reflect the global porosity value of the original binary mask (26.85%). Pore analysis was then performed on the reconstructed images using the analyze particles function in FIJI and the data retrieved from the reconstructions was compared to the pore information attained from Otsu’s method the original binary mask (Supplementary Figure S12a, b).


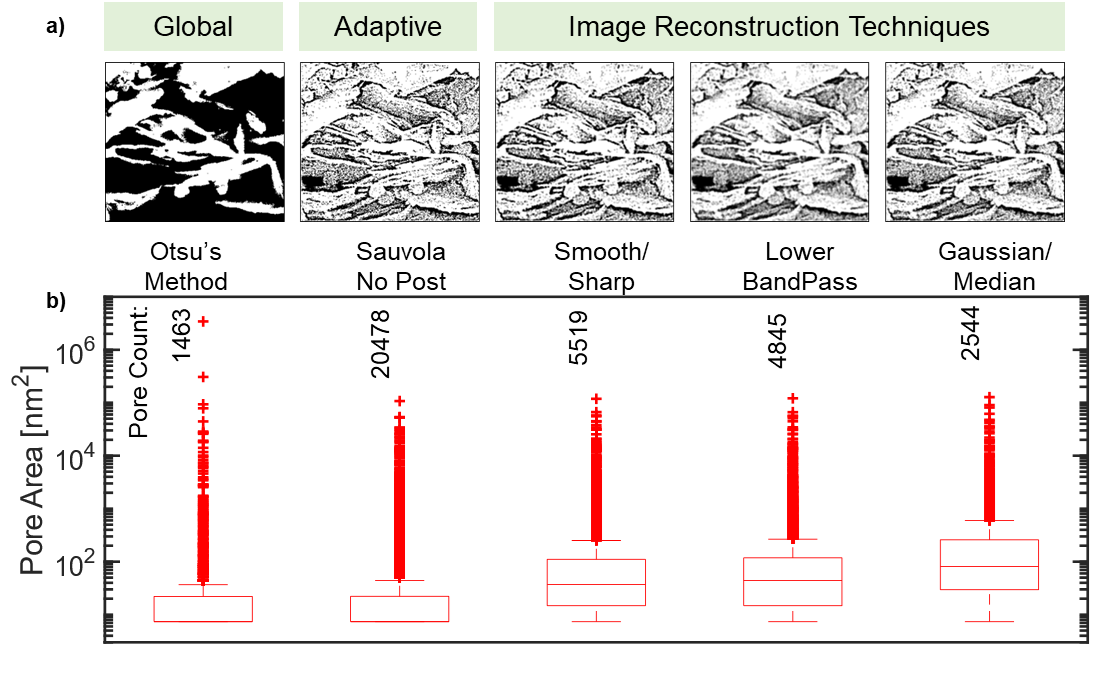


**Supplementary Figure S12. a**) The reconstruction techniques used for comparison against the automatic Otsu’s method and Sauvola’s method with no post-processing. **b)** statistical analysis of pores with various post-processing methods.


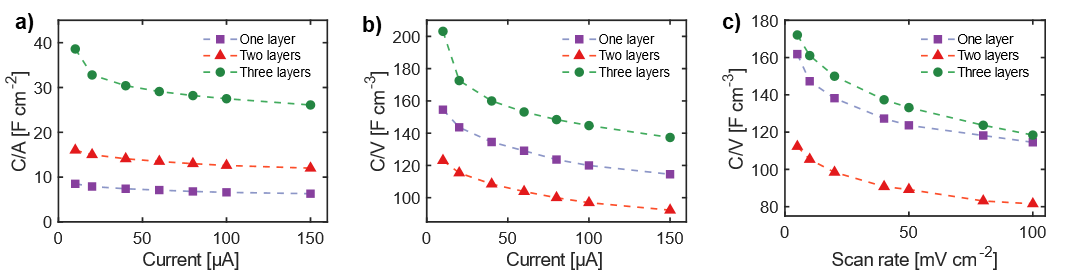


**Supplementary Figure S14. a)** C/A of printed MXene SCs at various current from 5 µA to 150 µA (calculated form GCD data). **b)** C/V of printed MXene SCs at various scan rates from 5 mV s^-1^ to 100 mV s^-1^ (calculated form CV data). **c)** C/V of printed MXene SCs at various current from 5 µA to 150 µA (calculated form GCD data).


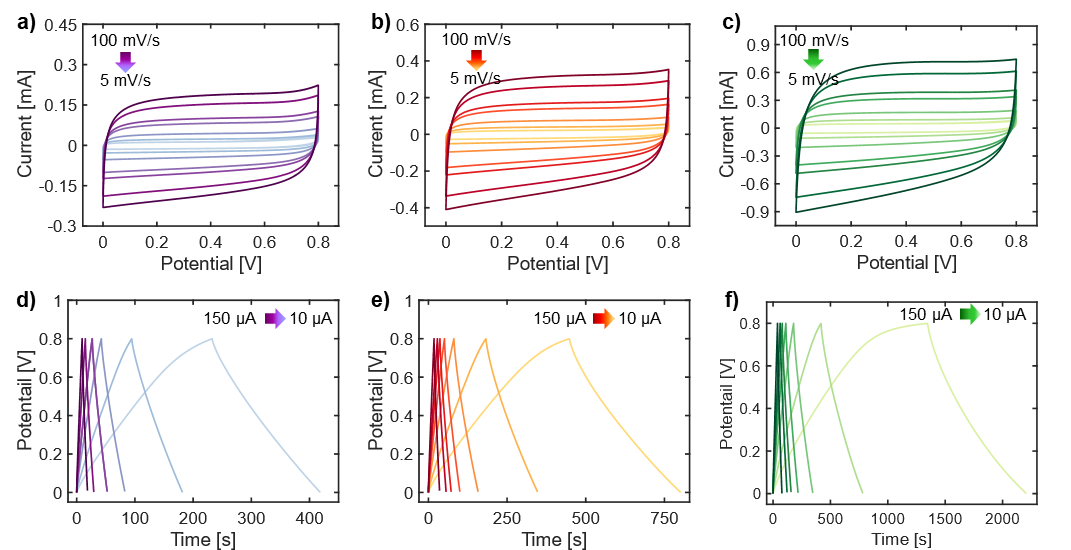


**Supplementary Figure S13. a-c)** CV curves at various scan rates of 5, 10, 20, 40, 50, 80, and 100 mV s^-1^ for one, two, and three printed passes, respectively. **d-f)** GCD curves at different current 10, 20, 40, 60, 80, 100, and 150 µA for one, two, and three printed passes, respectively.

The volumetric capacitances of the one printed passes MXene SCs have higher values than two printed passes, due to the variation in thickness. However, the areal capacitance for three passes are larger than two and two is larger than one printed pass. The three printed passes demonstrated the best electrochemical performance in terms of areal and volumetric capacitance*.*

The area on the CV curves at various scan rates does not show a lot of change (Supplementary Figure S15a). The potentionstate could not collect data for GCD test when the current increased more than 20 µA. Moreover, the GCD curves for 10 and 20 µA do not have a symmetrical shape, and there is a large voltage drop when the current is 10 µA. The EIS curve of the MXene electrode without gold shows a larger charge transfer resistance (R_ct_~8 kΩ), larger than printed MXene SCs with gold current collector (*R*_ct_~23 Ω), causing the decrease in the electrochemical performance of the device. Therefore, the gold current collector helps to reduce resistance and facilitate efficient charge/discharge processes, thereby enhancing the overall electrochemical performance of the supercapacitor.

**Supplementary Figure S15.** Electrochemical performance of printed Ti_3_C_2_T_x_ MXene ink without gold current collector **a)** CV, **b)** GCD, and **c)** EIS curves.


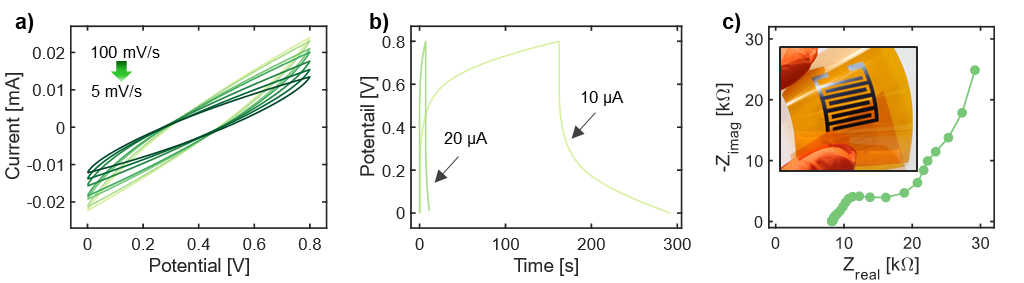

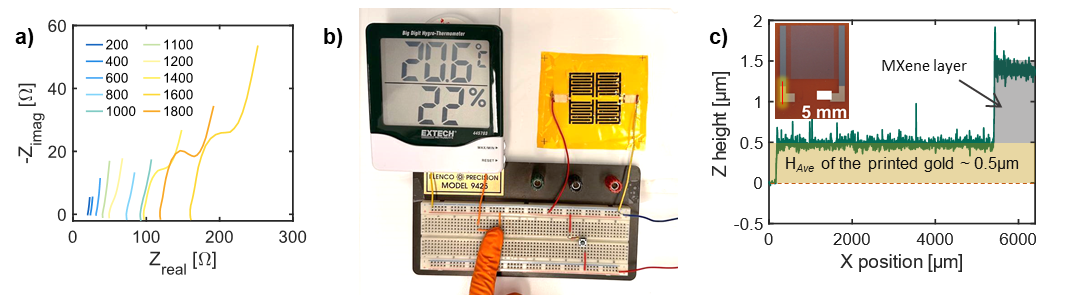


**Supplementary Figure S16. a)** Zoomed-in section of EIS data recorded every 200 cycle GCD from 200-1800 cycle GCD, highlighting the increase in resistance over charge-discharge cycles. **b)** Demonstration of a temperature-humidity sensor powered by integrated 2S2P MXene SCs, operating for approximately 30 s. Videos for this application can be found in the Supplementary Videos. **c)** Profilometry analysis on high-resolution MXene SCs for study the thickness of gold current collector. The analysis was conducted along the red line on MXene SCs, as shown in the inset, indicating a gold thickness of 0.5 µm.

**Supplementary Figure S17. a)** Optical image of high-resolution MXene printed. **b)** Profilometry analysis measuring the thickness of gold current collector along the orange line in (a). **c)** Profilometry data along the blue line in (a), showing the thickness of fingers for IDE structure, which includes both gold current collector and MXene active material. **d)** The distribution of the finger widths along the blue line (a), calculated from profilometry data. The average width of the fingers is measured to be 43.5 µm. **e)** CV curves recorded at various scan rates of 5, 10, 20, 40, 50, 80, and 100 mV s^-1^ for 2S2P integrated MXene SCs corresponding to (a). **f)** GCD curves at different current values of 10, 20, 40, 60, 80, 100, and 150 µA for the 2S2P integrated MXene SCs corresponding to (a).


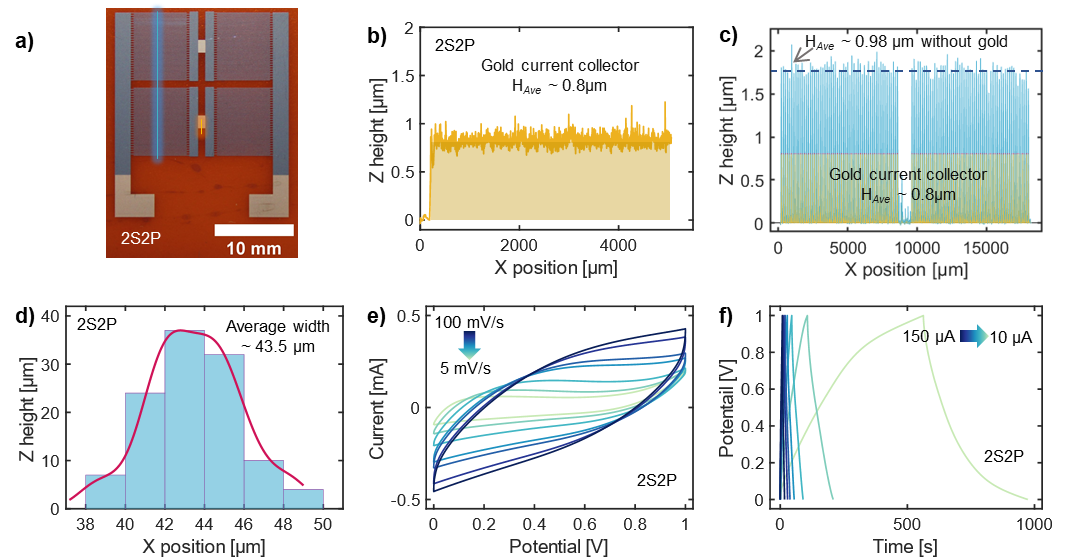


**Supplementary Figure S18.** Ragone plot of aerosol jet printed MXene SCs compared with previously reported supercapacitors. Detailed values can be found in Supplementary Table S5.


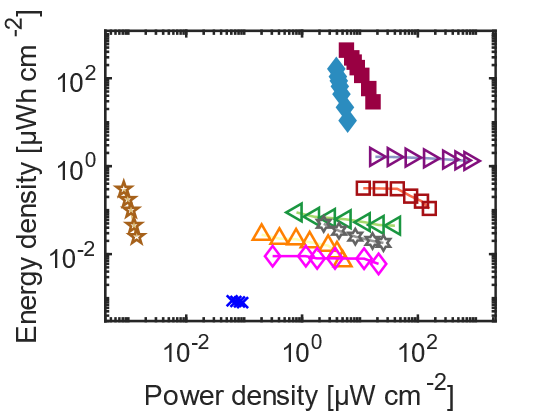


**Supplementary Tables**

**Supplementary Table S1.** Aerosol jet printing parameters for Gold, MXene ink, and dispersed
MXene in water.

| **Parameters** | **Gold** | **MXene ink** | **MXene ink HR printing** | **MXene in water** |
| --- | --- | --- | --- | --- |
| Nozzle diameter (µm) | 300 | 300 | 150 | 300 |
| Sheath gas flow (sccm) | 30-35 | 32-38 | 18-20 | 25-27 |
| UA atomizer flow (sccm) | 28-35 | 37-40 | 36-40 | 25-29 |
| UA atomizer current (mA) | 0.3-0.45 | 0.37-0.45 | 0.20-0.25 | 0.25-0.27 |
| Platen temperature (°C) | 65 | 50-55 | 50-55 | 50 |
| Water bath temperature (°C) | 25-30 | 30 | 25 | 25-30 |
| Process speed (mm/s) | 1-10 | 0.5-1 | 2-6 | 1 |
| Number of passes | 3 | 1-3 | 6 | 1 |

**Supplementary Table S2:** Resolution and gap study for additive manufacturing of MXene (Supplementary Figure S6 and S7)

**Supplementary Figure 7. (a)** and **(d)** CV curves for one and two printed passes. The scan rate varies from 5 mV/s to 100 mV/s. Both SCs show **(b)** and **(d)** GCD curves for one and three printed passes. **(c)** Areal capacitance of printed MXene SCs obtained from GCD curves. **(d)** Volumetric capacitance of printed MXene SCs obtained from GCD curves.

| **Materials** | **Method** | **Solvent system** | **Gap (µm)** | **Width (µm)** | **Ref.** |
| --- | --- | --- | --- | --- | --- |
| **Ti_3_C_2_T*_x_*** | **Aerosol jet printing** | **Water: Ethanol: Ethylene glycol**  **(5:4:1)** | **20** | **45** | **This Work** |
| Ti_3_C_2_T*_x_* | Inkjet printing | NMP | 50 | 130 | ^2^ |
| Ti_3_C_2_T*_x_* | Inkjet printing | Ethanol | 130 | 580 | ^2^ |
| Ti_3_C_2_T*_x_* | Inkjet printing | DMSO | 40 | 120 | ^3^ |
| Ti_3_C_2_T*_x_* | Inkjet printing | IPA | 50 | 100 | ^4^ |
| Ti_3_C_2_T*_x_*/PH1000 | Inkjet printing | Water:PH1000: glycol  (1:10:10) | 90 | 200 | ^5^ |
| Ti_3_C_2_T*_x_* | Thermal inkjet printing | Water | 250 | 600 | ^6^ |
| Ti_3_C_2_T*_x_*/C nanosphere | Aerosol jet printing | Water | 170 | 200 | ^7^ |
| Ti_3_C_2_T*_x_* | Extrusion | Water | 120 | 438 | ^2^ |
| Ti_3_C_2_T*_x_* | Extrusion | Water | 3 | 120 | ^8^ |
| N doped- Ti_3_C_2_T*_x_* | Extrusion | Water | 100 | 300 | ^9^ |
| Ti_3_C_2_T*_x_* | Screen printing | Water | 200 | 235 | ^10^ |

**Supplementary Table S3:** Areal capacitance comparison with previous SCs (Fig. 6i)

**Supplementary Figure 7. (a)** and **(d)** CV curves for one and two printed passes. The scan rate varies from 5 mV/s to 100 mV/s. Both SCs show **(b)** and **(d)** GCD curves for one and three printed passes. **(c)** Areal capacitance of printed MXene SCs obtained from GCD curves. **(d)** Volumetric capacitance of printed MXene SCs obtained from GCD curves.

| **Materials** | **Method** | **Electrolyte** | **C/A**  **(mF cm^-2^)** | **Ref.** |
| --- | --- | --- | --- | --- |
| **Ti_3_C_2_T*_x_*** | **Aerosol jet printing** | **NaClO_4_/PC** | **47** | **This Work** |
| **Ti_3_C_2_T*_x_*** | **Aerosol jet printing** | **PVA/H_2_SO_4_** | **122** | **This Work** |
| Ti_3_C_2_T*_x_* | Inkjet printing | PVA/H_2_SO_4_ | 12 | ^2^ |
| Ti_3_C_2_T*_x_* | Extrusion printing | PVA/H_2_SO_4_ | 43 | ^2^ |
| Ti_3_C_2_T*_x_*/PH1000 | Inkjet printing | PVA/H_2_SO_4_ | 22.6 | ^5^ |
| Graphene | Inkjet printing | PVA/H_3_PO_4_ | 1.25 | ^11^ |
| Electrochemical exfoliated graphene | Inkjet printing | Poly(4-styrenesulfonic acid) | 0.7 | ^12^ |
| Ti_3_C_2_T*_x_*/rGO | Spray coating+ laser cut mask | PVA/H_2_SO_4_ | 2.4 | ^13^ |
| Graphene/MXene (G-MX) | Spray coating | PVA/H_3_PO_4_ | 3.2 | ^14^ |
| Graphene/PEDOT | Spray coating | PVA/H_2_SO_4_ | 5.4 | ^15^ |
| PEDOT/MnO_2_ | Inkjet printing | PVA/LiCl | 0.26 | ^16^ |

**Supplementary Table S4:** Volumetric capacitance comparison with previous MXene SCs (Fig. 6j)

| **Materials** | **Method** | **Electrolyte** | **C/V**  **(F cm^-3^)** | **Ref.** |
| --- | --- | --- | --- | --- |
| **Ti_3_C_2_T*_x_*** | **Aerosol jet printing** | **NaClO_4_/PC** | **169** | **This Work** |
| **Ti_3_C_2_T*_x_*** | **Aerosol jet printing** | **PVA/H_2_SO_4_** | **611** | **This Work** |
| Graphene | Inkjet printing | PVA/H_3_PO_4_ | 37.2 | ^17^ |
| Ti_3_C_2_T*_x_*/rGO | Spray coating+ laser cut mask | PVA/H_2_SO_4_ | 80 | ^13^ |
| Graphene/MXene(G-MX) | Spray coating | PVA/H_3_PO_4_ | 33 | ^14^ |
| Graphene/PEDOT | Spray coating | PVA/H_2_SO_4_ | 27 | ^15^ |
| Electrochemical exfoliated graphene | Inkjet printing | Poly(4-styrenesulfonic acid) | 93.3 | ^12^ |
| Ti_3_C_2_T*_x_* | Spray coating+ laser cut mask | PVA/H_3_PO_4_ | 57.5 | ^18^ |
| Ti_3_C_2_T*_x_* | Scratch method | PVA/H_3_PO_4_ | 32.2 | ^19^ |
| Ti_3_C_2_T*_x_* | Inkjet printing | PVA/H_2_SO_4_ | 562 | ^2^ |
| Ti_3_C_2_T*_x_* | Laser cutting | PVA/LiCl | 5.78 | ^20^ |

**Supplementary Table S5:** Energy density and power density comparison with previous MXene SCs (Supplementary Figure S15)

| **Materials** | **Method** | **Electrolyte** | **E/A**  **(µWh cm^-2^)** | **P/A**  **(µW cm^-2^)** | **Ref.** |
| --- | --- | --- | --- | --- | --- |
| **Ti_3_C_2_T*_x_*** | **Aerosol jet printing** | **NaClO_4_/PC** | **16.77-5.82** | **29.12-436.82** | **This Work** |
| **Ti_3_C_2_T*_x_* (HR-MXene)** | **Aerosol jet printing** | **PVA/H_2_SO_4_** | **6.11-3.89** | **10.92-163.80** | **This Work** |
| Ti_3_C_2_T*_x_* | Extrusion printing | PVA/H_2_SO_4_ | 0.32-0.11 | 11.4-157.7 | ^2^ |
| Ti_3_C_2_T*_x_*/rGO | Spray coating+ laser cut mask | PVA/H_2_SO_4_ | 0.26-0.015 | 60-330 | ^13^ |
| Graphene/PEDOT | Spray coating | PVA/H_2_SO_4_ | 0.088-0.044 | 0.8-40 | ^15^ |
| Graphene | Spray coating | PVA/H_3_PO_4_ | 0.027-0.0069 | 0.2-5 | ^15^ |
| Ti_3_C_2_T*_x_* | Screen printed | PVA/H_2_SO_4_ | 1.64-1.32 | 18.4-778.3 | ^10^ |
| Graphene | Inkjet printing | PVA/H_3_PO_4_ | 0.00139-0.000833. | 0.025-0.3 | ^21^ |
| RuO_2_/PEDOT: PSS | Aerosol jet spraying | PVA/H_2_SO_4_ | 0.011-0.0015- | 19.47-0.28 | ^22^ |
| Graphene Q-dot | CVD-Shadow mask | PVA/H_3_PO_4_ | 0.00087-0.00079 | 0.062-0.094 | ^23^ |
| Transparent MXene | Spin-casting | PVA/H_2_SO_4_ | 0.049-0.018 | 25.37-2.36 | ^24^ |

**Supple****mentary Note**

**X-ray diffraction (XRD).** The crystal structure of powder Ti_3_AlC_2_ MAX phase and synthesized (*ml*)-Ti_3_C_2_T*_x_* MXene were characterized using powder X-ray diffraction (XRD) measurements conducted on a Rigaku Miniflex 600 X-ray diffractometer equipped with Cu K_α_ (λ_avg_=1.5418 Å) radiation source. The operating voltage was set to 40 kV, with a current of 15 mA. Specimens were scanned from 2θ= 5° to 90°, with a step size of 0.02° and a dwell time of 1 s.

**Scanning electron microscopy (SEM) and transmission electron microscopy (TEM).** To investigate the morphology of the Ti_3_AlC_2_ MAX phase, synthesized (*ml*)-Ti_3_C_2_T*_x_*, exfoliated Ti_3_C_2_T*_x_*, and printed MXene structures, scanning electron microscopy (SEM) was performed using FEI Teneo field-emission SEM. In addition, transmission electron microscopy (TEM) was performed using a JEOL-JEM-2100 HR analytical transition electron microscope operated at 200 kV to analyze the morphology and crystallinity of exfoliated Ti_3_C_2_T*_x_* MXene. TEM samples were prepared by drop-casting exfoliated Ti_3_C_2_T*_x_* nanosheet dispersion onto carbon-coated copper TEM grids. The samples were then fully dried in an Ar glovebox and loaded into the TEM sample holder to minimize the exposure to ambient air (less than 5 min). Energy dispersive X-ray spectroscopy (EDS) was also performed on the samples using an Oxford Instruments X-MAX 80 TLE spectrometer.

**X-ray photoelectron spectroscopy (XPS).** A Physical Electronics PHI Versa probe XPS system with a monochromated Al K_α_ X-ray source was utilized to collect spectra using a beam diameter of approximately 100 μm (25 W). For survey scans, a pass energy of 117.5 eV was used and evaluated from 0-1200 eV. The system was calibrated utilizing argon sputtered copper, silver, and gold as standards. Powdered samples were mounted by pressing the samples onto indium foil to minimize charging effects. For peak fitting, binding energies from previous reports were utilized with peak centers bound with a ±0.3 eV tolerance and a spin-orbit splitting Δ of 6.1 eV and 5.7 eV for MXene and TiO_2_ species, respectively, with the same bound tolerance^26^.The high-resolution core level spectra were deconvoluted and fitted using the OriginpPro software, and after subtraction of the background (Shirley).

**Atomic Force Microscope (AFM).** Exfoliated Ti_3_C_2_T_x_  nanosheets were imaged using the ScanAsyst PeakForce Tapping® Mode on a Dimension Icon Fastscan AFM (Bruker). ScanAsyst-Air-HR probes were used for producing the topography images and the image scan sizes ranged from 1 to 5 microns. Image processing and analysis was conducted using Nanscope Analysis V1.8 (Bruker). Topography maps underwent a flattening process to remove sample and tilt. The diluted dispersion of exfoliated Ti_3_C_2_T*_x_* in absolute ethanol was prepared and the solution was spin-coated on a silicon wafer and fully dried in an Ar glovebox. The lateral size histogram of the exfoliated MXene nanoflakes was obtained by measuring a sufficient number of flakes (~100) from obtained AFM images.

**Thermogravimetric analysis (TGA).** To evaluate the annealing condition after printing Ti_3_C_2_T*_x_* supercapacitor, thermogravimetric analysis (TGA) was performed using the Netzsch STA 449 F5 Jupiter simultaneous thermal analyzer. The Ti_3_C_2_T*_x_* ink was loaded into alumina (Al_2_O_3_) crucibles and left inside the oven at 50 °C for 2 h. Then TGA analysis was performed under argon gas, starting from 25 ℃ (room temperature) and reaching up to 1250 ℃, with a heating rate of 10 K min^-1^. Argon was used as both the purge and protecting gas, each with a flow rate of
20 mL min^-1^.

**Viscosity.** The viscosity of the formulated ink was determined using Brookfield Engineers Lab DVNext Cone rheometer with CP-40 spindle. 1 mL of MXene ink (concentration= 25 mg mL^-1^) was placed onto the stationary sample cone. The measurement was performed at room temperature, where the rotational speed was incrementally raised from 5 to 45 RPM. Each increment consisted of a 5 RPM increase, and each step was maintained for a duration of 30 s.

**Surface tension and contact angle.** The surface tension and contact angle of the formulated ink were determined using the Biolin Scientfic Attension Theta Lite tensiometer, employing the pendant drop and sessile drop techniques, respectively. To measure surface tension, a 5 μL droplet of ink was suspended from a pipette tip, and images were captured continuously for 60 s using a camera fixed on a homogeneously lit background that was generated from an LED light source. Subsequently, a 10 μL droplet of MXene ink was deposited onto a 1×1 cm^2^ gold square structure that was printed on Kapton. The contact angle was recorded for 10 s at room temperature, in an open-air environment and calculated by applying the classical Young-Laplace equation.

**Electrical Characterization.** To investigate the electrical properties of Ti_3_C_2_T*_x_* MXene, lines (2 mm×30 mm) were printed on glass substrate along with 5 gold contacts (1 mm×2 mm) for transmission line measurements (TLM). The number of passes for printing Ti_3_C_2_T*_x_* MXene was 1, 2, and 3, and the distance between the gold pad were varied from 2 to 6 mm, with increments of 1 mm between the gold pads. 2-point measurements were performed on the TLM structures to elucidate the electrical properties using a Keithley 4200 SCS/ Cascade Probe Station combo. Based on the collected results I-V curves were plotted and the sheet resistance of the lines with different pass number was converted to electrical conductivity based on the width and thickness of the printed lines using Ohm’s law. The total resistance ($R_{total})$ for each printed MXene was calculated from the slope of the corresponding I-V curves. From the total resistance ($R_{total})$ as a function of length of channel $(L, mm)$ (Figure 3i) four contact parameters can be extracted: sheet resistance ($R_{s}, Ω.cm )$, contact resistance ${(R}_{c}, Ω)$, sheet resistivity$(\rho_{s},Ω.cm )$ and conductivity $(\sigma, Sm^{-1} )$ based on linear transmission line model equation (1):

(1)

$$R_{total}=2R_{c}+\frac{R_{s}}{W}L$$

where $W$ is the width ($2 mm$) of the printed structure. The $R_{total}$ is the calculated total resistant from the I-V curves, $R_{c}$ is measured from the intercept of the straight line on the y-axis (Figure 3i). The slope of the linear fit yields ($\frac{R_{s}}{W})$, from which the sheet resistance is calculated as equation (2):

(2)

$$R_{s}=slope \times W$$

The sheet resistivity$(\rho_{s},Ω.cm )$ is then derived using the following equations (3-6):

(6)

(5)

(4)

(3)

$$R=\frac{\rho_{s}}{T}\times\frac{L}{W}$$

$$R=\frac{R_{s}}{W}L$$

$$R_{s}=\frac{\rho_{s}}{T}$$

$$\rho_{s}=R_{s}\times T$$

where T is the average thickness ($cm$) of the Ti_3_C_2_T*_x_* MXene lines which was determined using stylus profilometry as a function of the number of printed passes. The conductivity $(\sigma, Sm^{-1} )$ is calculated from the inverse of calculated sheet resistivity.

**Electrochemical Performance.** The areal capacitance (*C/A*, mF cm^-2^) of the two electrode systems was calculated based on the CV curves from the following equation (7):

(7)

$$\frac{C}{A}= \frac{2}{Av\Delta V}\int_{V_{1}}^{V_{2}} i \left( V \right)dV$$

where, *C/A* is the areal capacitance (mF cm^-2^), *A* is the total area of the two electrodes (cm^-2^), *v* is the scan rate (mV s^-1^), Δ*V* is the voltage window (*V*_2_-*V*_1_) and *i* represents the discharge current (mA).

Based on the CGD profiles, the areal capacitance (*C/A*, mF cm^-2^) of the printed MXene SCs was measured using equation (8):

(8)

$$\frac{C}{A}= \frac{2 i \Delta t}{A\Delta V}$$

where *i* represents the discharge current from GCD profiles (mA), *t* is the discharge time (s), *A* is the total area of the two printed electrodes (cm^-2^), and Δ*V* is the discharge voltage window (V).

The volumetric capacitance (*C/V*, F cm^-3^) of the MXene SCs was calculated based on the following equation:

(9)

$$\frac{C}{V}=\frac{C/A}{T}$$

where *T* is the film thickness (cm) of the printed device measured by the profilometry results of cross-section SEM of printed films.

For the Ragone plot, the areal energy density (*E/A*, µWh cm^-2^) and power density (*P/A*, µW cm^-2^) of the aerosol jet printed MXene supercapacitors were calculated based on the below equations (10) and (11):

(11)

(10)

$$E/A=\frac{1}{2} \times\frac{C}{A}\times\frac{\left( \Delta V \right)^{2}}{3.6}$$

$$P/A=\frac{E/A}{t} \times3600$$

where, *t* is the discharge time (s).

**Supplementary References**

1. Sauvola, J. & Pietikäinen, M. Adaptive document image binarization. *Pattern Recognit.* **33**, 225–236 (2000).

2. Zhang, C. (John) *et al.* Additive-free MXene inks and direct printing of micro-supercapacitors. *Nat. Commun.* **10**, 1795 (2019).

3. Vural, M. *et al.* Inkjet Printing of Self-Assembled 2D Titanium Carbide and Protein Electrodes for Stimuli-Responsive Electromagnetic Shielding. *Adv. Funct. Mater.* **28**, 1801972 (2018).

4. Jiang, X. *et al.* Inkjet-printed MXene micro-scale devices for integrated broadband ultrafast photonics. *npj 2D Mater. Appl.* **3**, 34 (2019).

5. Ma, J. *et al.* Aqueous MXene/PH1000 Hybrid Inks for Inkjet-Printing Micro-Supercapacitors with Unprecedented Volumetric Capacitance and Modular Self-Powered Microelectronics. *Adv. Energy Mater.* **11**, 2100746 (2021).

6. Uzun, S. *et al.* Additive-Free Aqueous MXene Inks for Thermal Inkjet Printing on Textiles. *Small* **17**, 2006376 (2021).

7. Wu, Y. *et al.* Aerosol Jet Printing of Hybrid Ti_3_C_2_T*_x_*/C Nanospheres for Planar Micro-supercapacitors. *Frontiers in Chemistry* **10**, 933319 (2022).

8. Shao, Y. *et al.* Room-temperature high-precision printing of flexible wireless electronics based on MXene inks. *Nat. Commun.* **13**, 3223 (2022).

9. Yu, L. *et al.* Versatile N-Doped MXene Ink for Printed Electrochemical Energy Storage Application. *Adv. Energy Mater.* **9**, 1901839 (2019).

10. Abdolhosseinzadeh, S. *et al.* Turning Trash into Treasure: Additive Free MXene Sediment Inks for Screen-Printed Micro-Supercapacitors. *Adv. Mater.* **32**, 2000716 (2020).

11. Sollami Delekta, S. *et al.* Fully inkjet printed ultrathin microsupercapacitors based on graphene electrodes and a nano-graphene oxide electrolyte. *Nanoscale* **11**, 10172–10177 (2019).

12. Li, J. *et al.* Scalable Fabrication and Integration of Graphene Microsupercapacitors through Full Inkjet Printing. *ACS Nano* **11**, 8249–8256 (2017).

13. Couly, C. *et al.* Asymmetric Flexible MXene-Reduced Graphene Oxide Micro-Supercapacitor. *Adv. Electron. Mater.* **4**, 1700339 (2018).

14. Li, H. *et al.* Flexible All-Solid-State Supercapacitors with High Volumetric Capacitances Boosted by Solution Processable MXene and Electrochemically Exfoliated Graphene. *Adv. Energy Mater.* **7**, 1601847 (2017).

15. Liu, Z. *et al.* Ultraflexible In-Plane Micro-Supercapacitors by Direct Printing of Solution-Processable Electrochemically Exfoliated Graphene. *Adv. Mater.* **28**, 2217–2222 (2016).

16. Wang, Y., Zhang, Y.-Z., Dubbink, D. & ten Elshof, J. E. Inkjet printing of δ-MnO_2_ nanosheets for flexible solid-state micro-supercapacitor. *Nano Energy* **49**, 481–488 (2018).

17. Li, L. *et al.* High-Performance Solid-State Supercapacitors and Microsupercapacitors Derived from Printable Graphene Inks. *Adv. Energy Mater.* **6**, 1600909 (2016).

18. Jiang, Q. *et al.* MXene Electrochemical Microsupercapacitor Integrated with Triboelectric Nanogenerator as a Wearable Self-charging Power Unit. *Nano Energy* **45**, 266–272 (2018).

19. Li, P. *et al.* Fabrication of high-performance MXene-based all-solid-state flexible microsupercapacitor based on a facile scratch method. *Nanotechnology* **29**, 445401 (2018).

20. Wang, N. *et al.* Laser-Cutting Fabrication of Mxene-Based Flexible Micro-Supercapacitors with High Areal Capacitance. *ChemNanoMat* **5**, 658–665 (2019).

21. Sollami Delekta, S., Smith, A. D., Li, J. & Östling, M. Inkjet printed highly transparent and flexible graphene micro-supercapacitors. *Nanoscale* **9**, 6998–7005 (2017).

22. Zhang, C. (John) *et al.* Highly flexible and transparent solid-state supercapacitors based on RuO_2_/PEDOT:PSS conductive ultrathin films. *Nano Energy* **28**, 495–505 (2016).

23. Lee, K. *et al.* Highly transparent and flexible supercapacitors using graphene-graphene quantum dots chelate. *Nano Energy* **26**, 746–754 (2016).

24. Zhang, C. (John) *et al.* Transparent, Flexible, and Conductive 2D Titanium Carbide (MXene) Films with High Volumetric Capacitance. *Adv. Mater.* **29**, 1702678 (2017).

25. Alhabeb, M. *et al.* Guidelines for Synthesis and Processing of Two-Dimensional Titanium Carbide (Ti_3_C_2_T_x_ MXene). *Chem. Mater.* **29**, 7633–7644 (2017).

26. Natu, V. *et al.* A critical analysis of the X-ray photoelectron spectra of Ti_3_C_2_T_z_ MXenes. *Matter* **4**, 1224–1251 (2021).
